# Defocused imaging-based quantification of plasmon-induced distortion of single emitter emission

**DOI:** 10.1038/s41377-023-01237-9

**Published:** 2023-09-18

**Authors:** Gwiyeong Moon, Taehwang Son, Hajun Yoo, Changhun Lee, Hyunwoong Lee, Seongmin Im, Donghyun Kim

**Affiliations:** 1 School of Electrical and Electronic Engineering, Yonsei University, Seoul, 03722 Korea; 2 Present Address: LG Innotek, Seoul, 07796 South Korea; 3 Present Address: Center for Systems Biology, Massachusetts General Hospital, Boston, MA USA; 4 Present Address: LG Display, Paju, Gyeonggi-do 10845 South Korea

**Keywords:** Imaging and sensing, Nanophotonics and plasmonics

## Abstract

Optical properties of single emitters can be significantly improved through the interaction with plasmonic structures, leading to enhanced sensing and imaging capabilities. In turn, single emitters can act as sensitive probes of the local electromagnetic field surrounding plasmonic structures, furnishing fundamental insights into their physics and guiding the design of novel plasmonic devices. However, the interaction of emitters in the proximity to a plasmonic nanostructure causes distortion, which hinders precise estimation of position and polarization state and is one of the reasons why detection and quantification of molecular processes yet remain fundamentally challenging in this era of super-resolution. Here, we investigate axially defocused images of a single fluorescent emitter near metallic nanostructure, which encode emitter positions and can be acquired in the far-field with high sensitivity, while analyzing the images with pattern matching algorithm to explore emitter-localized surface plasmon interaction and retrieve information regarding emitter positions. Significant distortion in defocused images of fluorescent beads and quantum dots near nanostructure was observed and analyzed by pattern matching and finite-difference time-domain methods, which revealed that the distortion arises from the emitter interaction with nanostructure. Pattern matching algorithm was also adopted to estimate the lateral positions of a dipole that models an emitter utilizing the distorted defocused images and achieved improvement by more than 3 times over conventional diffraction-limited localization methods. The improvement by defocused imaging is expected to provide a way of enhancing reliability when using plasmonic nanostructure and diversifying strategies for various imaging and sensing modalities.

## Abstract (web summary)

We investigate axially defocused images of a single fluorescent emitter near metallic nanostructure to explore distortion due to the emitter-localized surface plasmon interaction and retrieve information regarding precise emitter positions.

## Graphical abstract

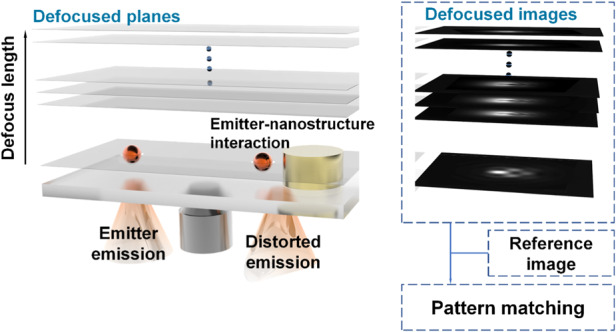

## Introduction

There has been a growing interest in detection and imaging of single molecules through use of plasmonic nanostructures^1–3^. Coupling of an incident electromagnetic wave to free electron in metal nanoparticles and nanostructure induces localized surface plasmon (LSP) and significant amplification of the field intensity with extreme confinement of light on a nanoscale^4^. This helps overcome the limitation of diffraction-limited light fields and a low signal arising from a single molecule by enhancing fluorescence intensity associated with Purcell factor^5^. In addition, plasmonic nanoparticle/structures were explored to enhance catalytic interactions^6^, optical trapping^7^, nonlinear optical response^8^, and super-resolution imaging^9–12^. On the other hand, single emitters have been utilized to characterize local fields and probe near-field distributions beyond diffraction limit. It was achieved by localization and intensity calibration of a single fluorescence emitter^13–15^ and measurement of Raman signal from single molecule^16–18^.

What is often disregarded in these applications is the distortion due to the interaction between fluorescent emitters and LSP. If the interaction becomes strong enough to significantly distort the acquired data, localization of a fluorescent emitter may contain error despite high measurement precision. Despite many unique advantages of single light emitters explored in many applications, recent studies regarding the detection and imaging of plasmonic light fields revealed that the point spread function (PSF) of a nanoparticle can be altered in close proximity to a plasmonic structure hindering precise fitting and localization of a single emitter^19–24^. A study of a fluorescent emitter near silver nanowires showed that the PSF forms two or multiple lobes rather than one clear spot of Airy function^19^. The orientation and the position of a dipole with regard to a metallic nanowire affect the PSF and create multi-lobed PSF, which a novel model was developed to fit and approximate with Hermite–Gaussian function^21^. Distortion of PSF may lead to mislocalization and therefore disparity between actual and apparent position of an emitter. The mislocalization was investigated by diverse approaches, e.g., including PAINT (point accumulation for imaging in nanoscale topography) microscopy^25,26^, dSTORM (direct stochastic optical reconstruction microscopy)^27^, alternate use of two light sources^28^, microfluidic devices^29^, and DNA origami^30,31^. The detected position of a single molecule was also found to be significantly different from the true position, while the direction of shift was either toward or away from the nanostructure^29,32^. This is caused by constructive and destructive interference between light emitted from emitters and nanostructure, the types of which depends on parameters such as emitter orientations, emitter-nanostructure distance, emission wavelength, and nanostructure morphology^29,32–34^. The way that the emission polarization state is affected by nanostructure was investigated by single-molecule polarization-resolved microscopy, i.e., the emission polarization can be rotated both toward and away from the localized SP mode of nanostructure, which may lead to mispolarization^35^. For overcoming mislocalization and mispolarization of an emitter, several approaches have been suggested by measuring lifetime^36^ and employing polarization-modulated single-molecule microscopy^37^. Furthermore, analytical analysis of a coupled dipole interaction model and numerical simulation using finite-difference time-domain (FDTD) were investigated to identify the underlying physics^32,35^.

In this work, we intend to estimate the extent of the distortion and, moreover, to check whether the distortion can be compensated by testing pattern-matching algorithm. To this end, we first fabricated plasmonic nanodisk arrays on a glass substrate and used fluorescent beads as well as quantum dots as a single emitter. The interaction between fluorescent emitters and plasmonic nanodisk arrays was investigated by monitoring three-dimensional PSF of emitters in the vicinity of a nanostructure, which is obtained from the defocused images. Defocused imaging of nanoscale emitters has been widely used for the measurement of radiation patterns^38–40^ and the determination of single molecule orientation^41^. A defocused image encodes the distance between an emitter and a plasmonic structure in the far-field radiation patterns, which can be conveniently acquired with high sensitivity over conventional analysis using focused images. Here, a defocused image of an emitter located near plasmonic nanostructure was acquired by moving and scanning an emitter on the structure toward the objective lens and compared to that of an emitter captured on bare glass. The distortion in the defocused pattern of a fluorescence bead or a quantum dot was confirmed. For quantum dot emitters, the defocused pattern was compared to single quantum-dot model based on three perpendicular linear dipoles with different emission strengths^39^. Then, the dipole position near the plasmonic nanodisk was estimated using pattern matching of defocused images. For this estimation, FDTD calculation was performed to obtain template images and the power flow of the emitter-nanodisk system while pattern matching algorithm was used to assessing similarity among template images. The algorithm, furthermore, was used to estimate the lateral position of an emitter by assessing similarity among template images.

The results described in this work provide a new approach to understand the interaction between single emitter and plasmonic nanostructure, based on which the detection and imaging performance of plasmonic nanostructures can be improved in practical applications.

## Results and discussion

### Defocused imaging of a fluorescent bead near nanodisk

**Fig. 1:**
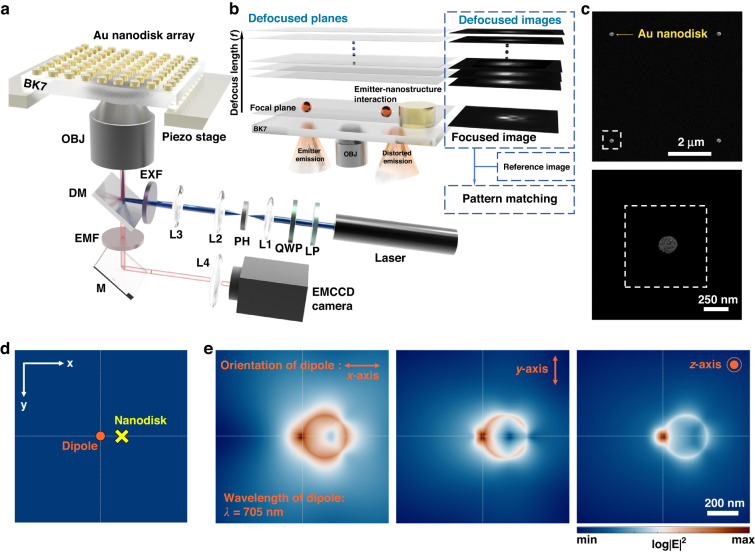
Defocused imaging for measurement of single emitter emission distorted by plasmonic nanostructure. **a** Schematic of the experimental optical set-up (OBJ objective lens, DM dichroic mirror, L1–L4 lens, M mirror, PH pinhole, LP linear polarizer, QWP quarter wave plate, EXF excitation filter, EMF emission filter, and Laser *λ* = 488 nm). **b** Conceptual schematic illustration: defocused patterns of an emitter in the vicinity of a nanodisk with defocus length (*f*) were acquired by moving emitter of the plasmonic substrate toward the objective lens. The acquired images are pattern-matched with a reference image obtained from FDTD calculation for distortion correction. **c** Scanning electron microscope image of nanodisk arrays with 5-μm period: (top) 2 × 2 nanodisk array and (bottom) a nanodisk magnified so that the dotted square corresponds to the top. **d** Schematics of two-dimensional area (*xy* plane) for FDTD simulation of near-field produced by a dipole emitter. The dipole was positioned at the center of the simulation area. Dipole and nanodisk position are marked with orange circle and yellow cross. **e** Calculated near-field intensity distribution produced by a dipole aligned in the *x*-, *y*-, and *z*-axis (*z* represents the depth axis). The dipole is located 10 nm away from the nanodisk edge. The nanodisk diameter and height were assumed to be 270 and 30 nm, respectively

The experimental optical set-up is illustrated in Fig. 1a, where the piezo stages allow axial scanning of the fluorescence of emitter. We measured defocused fluorescence images of a single emitter (fluorescent beads and quantum dots) located near gold nanodisk (see Fig. 1b for a schematic). The period of nanodisk arrays was set to be 5 μm, considering an enlarged nature of a defocused pattern. A scanning electron microscope image of a nanodisk array is presented in Fig. 1c. The near-field produced by a dipole in proximity to a nanodisk can be obtained using FDTD (Fig. 1d, “Methods”). Figure 1e shows the near-field distribution excited by a dipole aligned in the *x*-, *y-*, and *z*-axis (log scale in each plot for visualization). The near-field distribution can be utilized to obtain a defocused pattern of an emitter-nanodisk system (“Methods”).

**Fig. 2:**
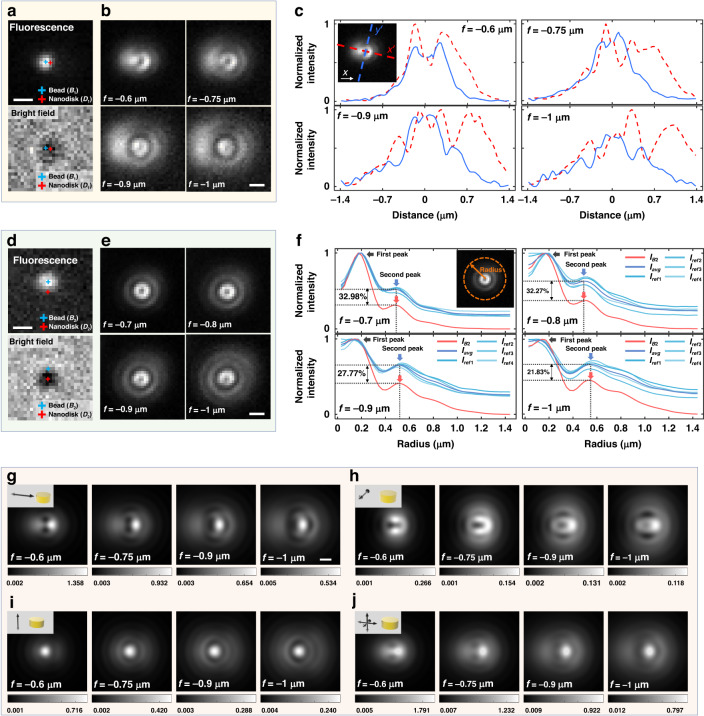
Distortion in defocused images of fluorescent beads near nanodisk. Two independent cases (1 and 2) of fluorescent beads (*B*_1_ and *B*_2_) near nanodisk (*D*_1_ and *D*_2_) are presented: case 1 (*B*_1_/*D*_1_) in **a**–**c** and 2 (*B*_2_/*D*_2_) in **d**–**f**. **a** Focused image of a fluorescent bead (*B*_1_, top) and bright field image of a nanodisk (*D*_1_, bottom). The center of fluorescence and bright field intensity was fitted by gaussian function (marked with blue and red cross). **b** Defocused images of *B*_1_ with a defocus parameter *f* = −0.6, −0.75, −0.9, and −1 μm. **c** Fluorescence intensity along the long axis (${x}^{{\prime} }$, horizontal) and the short axis (${y}^{{\prime} }$, vertical). Inset shows the axial definition. The intensity along the short axis presents high symmetry. **d** Image of a fluorescent bead (*B*_2_, top) and a nanodisk (*D*_2_, bottom) with the intensity center fitted by gaussian function (marked with blue and red cross). **e** Defocused images o*f B*_2_ with *f* = −0.7, −0.8, −0.9, and −1 μm. **f** Radial profile of fluorescence intensity of *B*_2_ and reference intensities in the absence of nanodisk (*I*_ref1_, *I*_ref2_, *I*_ref3_, and *I*_ref4_). *I*_avg_: average of the four reference intensity profiles. **g**–**i** Far-field defocused pattern of a dipole which is 20 nm away from the nanodisk was calculated. The dipole orientation assumed to be: **g** *x*-axis, **h** *y*-axis, and **i** *z*-axis. **j** Incoherent sum of a defocused pattern of three dipoles (oriented in the *x*, *y* and *z*-axis) for incoherent unpolarized source. Scale bars: 500 nm

Fluorescence beads (diameter *ϕ* = 40 nm) were randomly settled and immobilized on a nanodisk array sample by dropping and washing off the fluorescence bead solution. The whole field of fluorescence and bright-field images are presented in Fig. S1, in which some beads showing defocused patterns slightly different, due for example to the variation in the site of bead adsorption and the physical characteristics of a bead, were analyzed. A nanodisk (*D*_1_) and a fluorescent bead (*B*_1_) were imaged in the bright field and fluorescence image, respectively (Fig. 2a). The center position of a nanodisk and fluorescence intensity was obtained by Gaussian fitting and marked with red and blue symbols. For convenience, if we define *θ*_nf_ as the angle that the line connecting the centers of a fluorescent bead and a neighboring nanodisk makes against the *x*-axis, *θ*_nf_ = 170.8° in Fig. 2a. The position of *B*_1_ relative to *D*_1_ was localized by comparing physical properties with the AFM images presented in Fig. S2 and determined to be adsorbed to the left side of *D*_1_. A bump observed on the right in Fig. S2 may be associated with a quenched bead or a dust particle of a similar size. The effect of such a bump on the defocused images was checked with a model using dipoles aligned in multiple directions and shown to be negligible. *D*_1_ was also found to have a diameter of 295 nm. The disk size as well as the bead position was not directly measured by SEM because of charging effects of an electron beam. Defocused fluorescence images averaged over 10 sec of serial images with an exposure time of 0.1 s were taken by moving the focal plane. As a measure of defocus, we introduce *f* as a measure of defocusing, i.e., *f* = 0 represents an in-focus image. Because defocused images corresponding to a positive *f* suffer from poor contrast, we have only considered negative *f*, i.e., movement of an objective lens toward the sample relative to the focal plane. Defocused fluorescence images with *f* = −0.6, −0.75, −0.9, and −1 μm are presented in Fig. 2b. The negative sign denotes the movement of sample toward an objective lens. Note that the defocused patterns in Fig. 2b are elliptical without circular symmetry. This is in good contrast to the defocused patterns of fluorescent beads on the bare substrate shown in Fig. S3 that is observed with circular symmetry. The defocused patterns of Fig. 2b showed linear symmetry with respect to the line which connects the centers of a nanodisk and a fluorescence bead. This is confirmed by the intensity profiles of defocused fluorescence images along the horizontal and the vertical axis, as presented in Fig. 2c (horizontal and vertical axis shown in the inset of Fig. 2c). The results clearly suggest preferential symmetry along the vertical axis while not as symmetric horizontally, therefore elliptical defocused fluorescence images in Fig. 2b. The elliptical pattern is a result of distortion which is exacerbated by reduced SNR of defocused images of an emitter.

Consider another case of distortion in the defocused image of a fluorescent bead. Focused fluorescence and bright field image are shown in Fig. 2d, where *θ*_nf_ = 88.28°. Figure S4 shows the AFM images of nanodisk (*D*_2_) and fluorescent bead (*B*_2_). The diameter of nanodisk was about 355 nm, and the fluorescence bead (*B*_2_) was positioned on the nanodisk, unlike *B*_1_ which was beside the nanodisk. The defocused fluorescence images of *B*_2_ were obtained with *f* = −0.7, −0.8, −0.9, and −1 μm (Fig. 2e). The images showed the radial symmetry as those of fluorescence beads on the bare substrate. However, there was a significant difference in the intensity fluctuation in the radial direction. The radial intensity profiles of *B*_2_ (*I*_*B*2_) and reference beads on the bare substrate (*I*_ref1,_ *I*_ref2,_ *I*_ref3,_ and *I*_ref4_) were plotted in Fig. 2f. The average intensity distribution (*I*_ref_,_avg_) was also calculated with four reference intensity profiles (*I*_ref1,_ *I*_ref2,_ *I*_ref3,_ and *I*_ref4_). The intensity profile of *I*_*B*2_ and *I*_ref_,_avg_ have two intensity peaks at similar locations, where the first peak is located near 0.2 μm and the second peak at 0.49–0.55 μm. The peaks represent an intensity maximum of defocused rings. The peak position and the intensity distribution of the defocused pattern are determined by diverse parameters including the degree of defocus (*f*), refractive index of sample/immersion medium, and NA of an objective lens. In Fig. 2f, we attempted to capture changes in the peak position and the intensity arising from the interaction with a nanodisk while these parameters are fixed. The intensity of the first peak was identical. In contrast, the second peak of intensity values in *I*_*B*2_ and *I*_ref_,_avg_ showed disparity by about 21.8–33.0%, which represents the distortion by the nanodisk in the defocused patterns of *B*_2_. The reason why the peak location and intensity of the defocused pattern *B*_2_ can be directly compared with a bead on the bare substrate is that *B*_2_ showed circular symmetry. However, *B*_1_ presents a different case, where the circular symmetry is broken with distortion, as shown in Fig. 2c. The distinct trend of distortion observed in *B*_1_ and *B*_2_ is attributed to the relative position of an emitter to the nanodisk, i.e., *B*_1_ is located at the side of the nanodisk (*D*_1_), whereas *B*_2_ is on the top of *D*_2_. Note also that the distortion in the case of *B*_1_ manifests itself with directional variation because specific intensity profiles depend on various experimental parameters such as *f* and the site of adsorption of fluorescence beads, some of which are difficult to control. For *B*_2_, the distortion is isotropic, yet local variation may cause azimuthally non-uniform artifacts in the data despite overall circular symmetry.

### Theoretical understanding

Numerical simulation was performed using FDTD to obtain defocused images of a dipole for comparison with experimental results. Simulation parameters reflect the experimental setting presented in Fig. 2a–c to emphasize an asymmetric bead-plasmon interaction when a bead is adsorbed to the side so that the distortion is significant: therefore, the diameter and height of a nanodisk were set to be 300 and 30 nm. An electric dipole was placed 20 nm away from the nanodisk and substrate. From the simulations of a dipole oriented along the *x*, *y*, and *z* axis with *f* = −0.6, −0.75, −0.9, and −1 μm. Defocused far-field images of the dipole were obtained in the vicinity of the nanodisk and presented in Fig. 2g–i. Note that *x*-axis was configured to be parallel to the direction of a vector which connects dipole to nanodisk. In each image, intensity scales were adjusted for effective visualization. Significant disparity was observed between simulated and reference defocused patterns of a dipole on bare substrate (Fig. S5). The reference defocus patterns of a dipole oriented in parallel to the optical axis (*z*-axis) show radial symmetry, while those of a dipole perpendicular to the optical axis exhibit double-linear symmetry with respect to the horizontal and vertical line. In contrast, a dipole located near a nanodisk shows a completely different single-axis symmetry with respect to the horizontal line, as presented in Fig. 2g–i, for all three orientations. Note that a dipole along the *x*-axis produced higher intensity than those in the other directions. The maximum and average intensity of each defocused image of Fig. 2g–i are plotted in Fig. S6: *I*_max,*x*_/*I*_max,y_ ~ 5 between dipoles oriented in the *x* and *y*-axis and *I*_max*,x*_/*I*_max*,z*_ ~ 2 between dipoles oriented in the *x*- and *z*-axis for *f* = −0.6 to −1 μm. Energy transfer between a dipole and a nanodisk affects and varies the far-field intensity depending on many parameters that include dipole orientation and dipole-nanodisk distance. More details are provided in section “Far-field distribution of a dipole near nanodisk.” The defocused far fields of dipoles in three orientations were superposed incoherently to simulate a fluorescence bead as an incoherent unpolarized dipole source, as shown in Fig. 2j. The defocused images, when directly compared with Fig. 2b, are in excellent agreement with the experimental result.

### Defocused imaging of a quantum dot near nanodisk

**Fig. 3:**
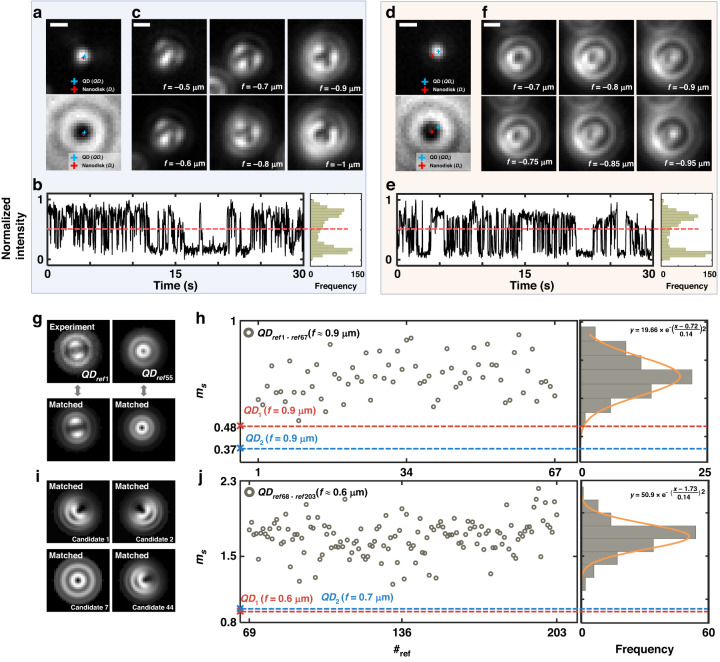
Distortion in defocused images of quantum dot near nanodisk and assessment of distortion using pattern matching. Two independent cases (1 and 2) of quantum dots (QD_1_ and QD_2_) near nanodisk (*D*_3_ and *D*_4_) are presented: case 1 (QD_1_/*D*_3_) in (**a**–**c**) and 2 in (**d**–**f**) (QD_2_/*D*_4_). **a** Fluorescence image of a quantum dot (QD_1_, top) and bright field image of a nanodisk (*D*_3_, bottom). The center of fluorescence and bright-field image is marked with blue and red cross. **b** Time-lapsed normalized fluorescence intensity fluctuation of QD_1_. Histogram of the normalized fluorescence intensity (right). **c** Defocused images of QD_1_ while *f* is varied. **d** Image of a quantum dot (QD_2_, top) and a nanodisk (*D*_4_, bottom). **e** Time-lapsed normalized fluorescence intensity fluctuation of QD_2_. Histogram of the normalized fluorescence intensity (right). **f** Defocused images of QD_2_ **g** Pattern matching of a reference quantum dot. **h** Similarity index (*m*_s_) for the defocused pattern of QD_1_, QD_2_, and reference quantum dots (QD_ref1–ref67_) with *f* = −0.9 μm. **i** Pattern matching of QD_1_. **j** *m*_s_ for the defocused pattern of QD_1_ (*f* = −0.6 μm), QD_2_ (*f* = −0.7 μm), and reference quantum dots (QD_ref1–ref67_). Scale bars: 500 nm

Defocused images of a quantum dot near nanodisk were measured. Quantum dots were randomly settled and immobilized to a nanodisk array sample by dropping quantum dot solution and washing off the solution with distilled water, as in the case of bead imaging. The whole area of quantum dot emission with a bright-field image of a nanodisk array is presented in Fig. S7. A fluorescence image of a quantum dot (QD_1_) in close proximity to a nanodisk (*D*_3_) and its bright-field image are shown in Fig. 3a. Both images were obtained by averaging 100 frames taken with an exposure time of 0.1 s. The center position of *D*_3_ and QD_1_ was obtained by gaussian-fitting bright-field and fluorescence image, respectively (marked with red and blue cross symbol in Fig. 3a). The distance between the center of *D*_3_ and QD_1_ was 47.8 nm, which may differ from the actual distance due to mislocalization, while *θ*_nf_ = 59.18°. Figure S8 presents AFM images of a nanodisk: the diameter and height were determined to be 260 nm and 30 nm. We have also assessed whether QD_1_ is a single quantum dot or aggregation by measuring single quantum dot characteristics of distinct two-level blinking on/off states^42^. The fluorescence intensity of a quantum dot was calculated from a focused image sequence of 1000 frames with 0.03-s frame rate and plotted in Fig. 3b. The intensity plot showed the two-level on/off state demonstrating characteristics as a single quantum dot, which can also be confirmed in the intensity histogram. The defocused fluorescence images of QD_1_ presented in Fig. 3c were averaged over 100 s in series with an exposure time 1 s while moving the focal plane corresponding to *f* = −0.5, −0.6, −0.7, −0.8, −0.9, and −1 μm. Note that the distortion of quantum dot emission by a nanodisk may be ambiguous in contrast to the case of fluorescent beads evident in Fig. 2b, e because the defocused images of quantum dots depend on the orientation of emission dipoles and the ratio of emission strength of orthogonal dipoles. This allows the formation of a variety of images, as presented in Fig. S9, while the images are identical for fluorescent beads with isotropic emission (see in Fig. S10)^39,40,43^. In this work, pattern-matching algorithm is used to determine whether quantum dot emission is affected by a nanodisk by comparing experimental defocused images with single quantum dot modeling.

Another case of a quantum dot emitter (QD_2_/*D*_4_) is considered in Fig. 3d–f which shows defocused images of QD_2_ near a nanodisk *D*_4_. In this data set, the distance between the center of *D*_4_ and QD_2_ was obtained as 225.5 nm (see Fig. S11 for AFM images with the diameter and height of a nanodisk at 267 nm and 30 nm). Also shown in Fig. 3e that QD_2_ was a single quantum dot showing two-level on/off states. The defocused fluorescence images of QD_2_ with *f* = −0.7, −0.75, −0.8, −0.85, −0.9, and −0.95 μm are presented in Fig. 3f, which again shows the effect of anisotropic emission of QD_2_ near *D*_4_.

Experimentally obtained defocused images were matched with simulation of a single quantum dot to understand the nature of quantum dot emission near a nanodisk. If affected by a nanodisk, a defocused pattern is not expected to match the patterns of a single quantum dot, while it should match well on the bare substrate. Note that a defocused image of a single quantum dot on the bare substrate can be analytically obtained by calculating energy flux (“Methods”). For the matching, a single quantum dot was modeled with a normalized superposition of three orthogonal linear dipoles. Far-field images were then obtained by calculating Poynting vector and intensity distribution produced by each dipole (“Methods”)^39^. The template for image matching was acquired by scanning dipole orientations as well as the ratio of emission strength among orthogonal dipoles. The parameters for the calculation and subsets of template images can be found in Table S1 and Fig. S12. An image in the template which minimizes least-square error with experimental image data was defined as a matched pattern. We have also used similarity index *m*_*s*_, which is described in “Methods,” to evaluate image similarity quantitatively. For comparison, 203 experimental defocused images of a quantum dot on the bare substrate with *f* $\approx$ −0.9 μm (QD_ref1–67_) and −0.6 μm (QD_ref68–203_) were obtained (the images provided in Fig. S9 and S13). Two examples of experimental and matched images are in excellent agreement as presented in Fig. 3g. The distribution and histogram of *m*_s_ for QD_ref1–67_ and QD_ref68–203_ are shown in Fig. 3h, j with an average at 0.72 and 1.73, respectively. On the other hand, defocused images of QD_1_ and QD_2_ in the vicinity of a nanodisk did not match well with the template images. As an example of poor matching, Fig. 3i shows matched images of 1st, 2nd, 7th, and 44th lowest least square error when matched with a defocused image of QD_1_ (*f* = −0.9 μm). The poor matching arises from defocused images of QD_1_ not being included in the single quantum dot template image. Note also that *m*_s_(QD_1_) = 0.48 and *m*_s_(QD_2_) = 0.37 at *f* = −0.9 μm, while *m*_s_(QD_1_) = 0.92 and *m*_s_(QD_2_) = 0.95 at *f* = −0.6 μm. Overall, similarity observed with QD_1_ and QD_2_ is much lower than the case of reference QDs (QD_ref1–203_). These results imply that defocused images of a quantum dot near a nanodisk cannot be modeled with a single quantum dot dipole model, while confirming that emission of an emitter is affected by the nanodisk.

### Far-field distribution of a dipole near nanodisk

**Fig. 4:**
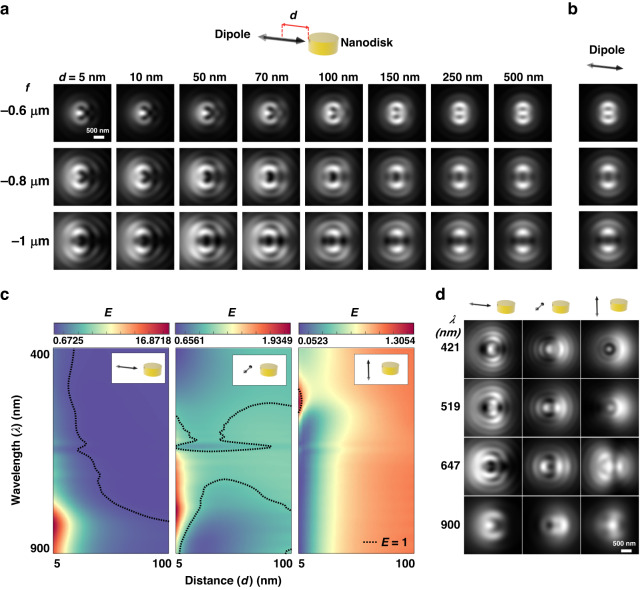
FDTD calculation of far-field of dipole near nanodisk. **a** Far-field defocused image calculated with *f* = −0.6, −0.8, and −1 μm of a dipole along the *x*-axis as the distance (*d*) between nanodisk and dipole is varied from 5 to 500 nm. **b** Defocused image of a dipole in the *x*-axis (*f* = −0.6, −0.8, and −1 μm) on bare glass. **c** Radiated power enhancement of three orthogonal dipoles in the vicinity of a nanodisk in terms of distance (*d*) and wavelength (*λ*). Black dotted lines represent a contour with radiated power enhancement *E* = 1. **d** Defocused images of three orthogonal dipoles at *λ* = 421, 519, 647, and 900 nm

FDTD calculation was carried out to obtain the defocused image of a quantum dot near a nanodisk. Figure 4a shows the far-field defocused images of a dipole along the *x*-axis (*λ* = 705 nm) near a nanodisk with 270-nm diameter and 30-nm height, while the distance (*d*) between nanodisk edge and dipole was varied from 5 to 500 nm. With *d* > 150 nm, the defocused images of a dipole were almost identical to those on bare substrate presented in Fig. 4b. On the other hand, defocused images vary noticeably as *d* decreases below 100 nm, in which case it would be challenging to estimate the dipole orientation from defocus images. More cases of other dipole orientations are provided in Fig. S14. The results, in other words, indicate that the defocused images of a dipole are highly dependent on the dipole orientation as well as the distance *d* near a nanodisk.

For more in-depth analysis of dipole-nanodisk interactions, the power radiated into the far-field was obtained for a dipole positioned near a nanodisk ($${P}_{R}$$) and a dipole on the bare substrate ($${P}_{R}^{0})$$ using FDTD method. The radiated power enhancement was defined as $$E={P}_{R}/{P}_{R}^{0}$$ and calculated for each of the three orthogonal dipole orientations with *d* = 5–100 nm and *λ* = 400–900 nm, as shown in Fig. 4c (see Fig. S15 for more details)^39^. Contour on which *E* = 1 is plotted in black dotted lines in each plot of Fig. 4c. *E* converges to 1 as *d* increases for all three dipole orientations in agreement with defocused images of a dipole with large *d* that are almost identical to those of a dipole on the bare substrate. Among the three orientations, a dipole in the *x*-axis represents higher radiative power enhancement than dipoles along the other directions. The wavelengths that produce the highest *E* vary for the three dipole orientations because of anisotropicity of dipole-nanodisk structure. Note that only two dipole orientations (parallel and perpendicular to surface) are needed when a dipole is isotropic, e.g. nanosphere^44,45^. Figures 4d and S16 show defocused images of a dipole in three orthogonal orientations for *λ* = 421–900 nm at *d* = 10 nm. If an emitter is not adsorbed to the surface, the gap between the dipole emitter and the surface may also influence far-field radiation patterns, although this may be alleviated in motion. It was found that the emission wavelength of a dipole has a significant impact on the formation of defocused images of the dipole-nanodisk system.

### Effectiveness of the coupled dipole model

**Fig. 5:**
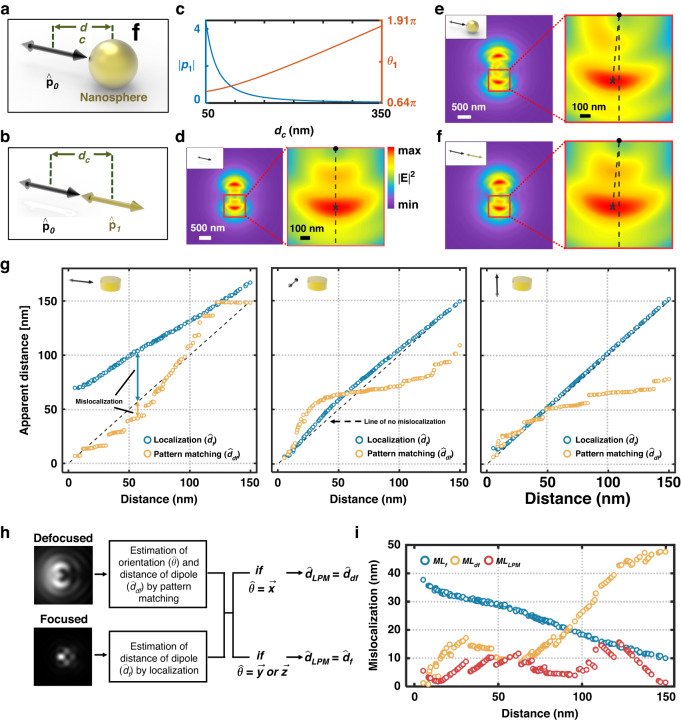
Coupled dipole model for distortion of defocused images and pattern matching for estimation of position of dipole near nanodisk. **a** Schematic of a dipole ($\vec{{p}_{0}})$ near a nanosphere. **b** Schematic of a two-dipole model: $\vec{{p}_{0}}$ interacts with a dipole $\vec{{p}_{1}}$, which replaces the nanosphere in (**a**). **c** Amplitude and phase of $\vec{{p}_{1}}$. Simulated defocused image of a dipole: **d** on bare substrate and **e** in the vicinity of a nanosphere. **f** Defocused image of a two-dipole model at *f* = −1.2 μm. **g** Estimation of distance between dipole and nanodisk based on defocused (orange circle) and conventional (blue circle) fluorescence images. Black dashed line represents *y* = *x*. **h** Procedure of distance estimation for the complementary localization and pattern-matching (LPM) approach. **i** Mislocalization of a dipole near nanodisk estimated by conventional localization-based method (*ML*_*f*_), pattern-matching with defocused images (*ML*_*df*_), and LPM (*ML*_*LPM*_)

The distortion in defocused images by nanostructure can also be described analytically with a coupled dipole model. The model describes an emitter-nanoantenna system by approximating it as two coupled and interfering dipoles after assuming a nanoantenna as a polarization induced dipole^32^. Consider a dipole ($\vec{{p}_{0}}$) used to model a fluorescent emitter and positioned at a distance 50 nm from the center of gold nanosphere (diameter: 80 nm). Higher-order multipole modes may contribute to the resonance characteristics, which we have not included in the current model because the higher-order effects may be weaker than the dipole modes in the wavelength range^46,47^. We used a nanosphere in the model for ideal and simple calculation. In one case, a dipole is placed in proximity to a gold nanosphere (Fig. 5a). In the other case, the gold nanosphere is replaced with another dipole ($\vec{{p}_{1}}$) positioned at the nanosphere center (Fig. 5b). For simplicity, $\vec{{p}_{0}}$ was assumed as a unit vector. The dipoles were assumed to couple in the parallel alignment which can be dominant in the experimental setting^48^. The amplitude and phase of $\vec{{p}_{1}}$ was calculated based on the coupled dipole model (see Text S1 in Supplementary Information for details)^32^, as presented in Fig. 5c. The amplitude of $\vec{{p}_{1}}$ decreases toward zero as the distance (*d*_c_ in Fig. 5a, b) increases up to 350 nm. The phase was also affected by the dipole–dipole distance which can lead to constructive and destructive interference with $\vec{{p}_{0}}$^29,32^. As a reference, a defocused far-field image (*f* = −1.2 μm) of a dipole on bare substrate was obtained using FDTD (Fig. 5d), while the images corresponding to the two cases are presented in Fig. 5e, f. The defocused images of the two cases are similar, where the angle that the line connecting the center of pattern (marked as dot) and maximum intensity position (marked as ×) against the vertical line was found to be identical at 5.59° in both cases (magnified images on the right of Fig. 5e, f). The result implies that the coupled two-dipole model can provide insights on the distortion of the defocused images with a dipole in the proximity of a nanosphere.

### Estimation of true position of a dipole

We now investigate the feasibility of use of defocused images for estimation of true positions of a dipole, which may approximate an emitter if three orthogonal dipoles are superposed in the 3D space, in the presence of distortion. For the estimation, pattern matching between target and template image was carried out. The process of finding the true position of a dipole in the lateral plane is detailed in Fig. S17. For simplification, dipoles aligned in the three principal axes (*x*-, *y-*, and *z*-axis) were considered for template and target images. Detailed parameters including the distance and wavelength to obtain template and target images are provided in Fig. S17. Note that Poisson noise was added to the target image (pixel size: 80 nm) to simulate experimental conditions. The result of estimation obtained from matching defocused images (*f* = −0.9 μm) was compared to the conventional method based on localization of focused images. The estimated distance between dipole and nanodisk of the two methods are presented in Fig. 5g. The estimated distance by pattern matching of defocused images and localization of focused images are denoted as $\hat{d}$_df_ and $\hat{d}$_f_, respectively. Clearly, the performance of estimation between the two approaches depends on the dipole orientation and the distance (*d*). In the case of a dipole in the *x*-axis, it was found that the estimation based on pattern matching of defocused images performed far better than based on conventional focused fluorescence images. On the other hand, this was not the case with dipole in the *y-* and *z*-axis, likely due to lower sensitivity of defocused images to the distance than a dipole in the *x*-axis. This argument can be confirmed with a structural similarity index measure (SSIM)^49^, a useful metric for assessment of image similarity, between defocused images of *d* < 150 nm and reference (*d* = 150 nm), which suggests that defocused images of a dipole in the *x*-axis are more sensitive to the distance with lower SSIMs than dipole in the *y* and *z*-axis. (Fig. S18).

For estimation of distance, we use complementary localization and pattern matching (LPM), which employs the two methods in tandem by taking $\hat{d}$_df_ for a dipole in the *x*-axis and $\hat{d}$_f_ for those in the *y* and *z*-axis (Fig. 5h). Note that the orientation of a dipole can be estimated by pattern matching of defocused images for *d* < 150 nm (Fig. S19). The mislocalization in the case of three orthogonal dipoles, which may be induced by localization of focused images ML_f,(*x*,*y*,*z*)_, pattern matching of defocused images ML_df,(*x*,*y*,*z*)_, and LPM ML_LPM(*x*,*y*,*z*)_, was calculated from the estimated distance (Fig. S20). Figure 5i presents dipole-averaged mislocalization, i.e., $\sqrt{{{{ML}}_{x}\left(d\right)}^{2}+{{{ML}}_{y}\left(d\right)}^{2}+{{{ML}}_{z}\left(d\right)}^{2}}$ obtained with each method. In contrast to the conventional localization-based method, mislocalization that may be caused by the pattern-matched estimation increases with distance because defocused images do not change significantly at a large distance by converging to images of a dipole in the absence nanostructure (see Fig. 5i and S18). In terms of an averaged mislocalization over *d* < 150 nm, LPM achieved the lowest precision of 7.01 nm, which is 2.90 and 3.41 times better than what may be achieved with pattern matching and localization-based method (20.42 and 23.91 nm of mislocalization, respectively). Although disparity may arise in the results due to the geometrical differences, the estimation based on the coupled dipole model reflects the experimental conditions and thus sheds light on the performance of LPM to the first degree.

## Conclusion

In this work, we demonstrated that three-dimensional point spread function of a single emitter can be significantly distorted by nanostructure. We measured defocused images of fluorescence beads and quantum dots in the vicinity of nanostructure and compared the images with those in the absence of nanostructure. It was straightforward to notice that defocused pattern of fluorescence beads was distorted, although the ground truth of fluorescence emitters was not precisely determined due to the experimental limitation, i.e., precise characterization of the gap between nanoparticles and nanodisk is difficult with AFM. Pattern matching algorithm was utilized using a dipole model that approximates an emitter to demonstrate indirectly that defocused pattern of quantum dot close to nanodisk was not fit to a single quantum dot and distorted. Future work will focus on the spatial localization of a nanoparticle using imaging methods with higher resolution, such as SEM, for comparison with quantified values calculated from defocused images. Furthermore, precise control of distance between nanostructure and an emitter using chemical binding will be desired: for instance, utilization of biotin-PEG linker functionalized nanostructure and streptavidin conjugate fluorescence emitters with the distance adjusted by the PEG length. Moreover, pattern-matching of a defocused pattern was employed as novel strategy to estimate the lateral position of a dipole near nanodisk, while it can be potentially extended to combine with localization method based on focused fluorescence images. Ultimately, these methods provide understanding of interaction between an emitter and nanostructure and open a new way for engineering of nanostructure inducing light-matter interaction.

## Methods and materials

### Nanodisk fabrication

To understand the interactions with single emitters, nanodisk arrays were fabricated first by cleaning glass coverslip using acetone, isopropyl alcohol, and diluted water with sonication. After spin-coating positive resist (AR-P 679.02, Allresist, Strausberg, Germany) at 4000 rpm and conductive polymer (AR-PC 5091.02, Allresist, Strausberg, Germany) at 3000 rpm, nanodisk patterns were defined by electron beam lithography. Lift-off process was conducted by depositing 30-nm-thick gold film with a 1-nm chromium adhesion layer and removing resist. Gold nanodisk arrays of 30-nm height with a period Λ = 5 μm were created in the diameter range from 225 to 335 nm. Characterization of fabricated nanodisks was performed with SEM (Vega3, Tescan, Brno, Czech Republic) and AFM (XE7, Park Systems, Suwon, Korea).

### Numerical field calculation

Electromagnetic simulation using three-dimensional FDTD was performed to calculate near-field distribution and far-field patterns produced by a dipole–nanodisk interaction. The dipole wavelength was set to be *λ* = 645 nm and 705 nm to simulate fluorescence beads and quantum dots. Dielectric function of gold was obtained from Palik^50^. Far-field images of a dipole were obtained by propagating simulated near field distribution assuming an objective lens with NA = 1.49. A near-field monitor was positioned in the glass medium below the dipole. Grid size of a mesh was set to 2.5 nm.

### Optical set-up

For experimentation, an inverted microscope (IX-73, Olympus, Japan) equipped with a 1.49-NA, ×100 oil immersion objective (Olympus, UApoN oil immersion TIRF lens, NA 1.49) was used. Wide-field illumination was accomplished using 488 nm laser (Obis 488 LS, Santa Clara, CA, USA) as light source. A quarter wave plate was inserted to achieve circular polarization for imaging quantum dots. An EMCCD (iXon Ultra 897, Andor) was employed to measure fluorescence of polystyrene beads (TransFluoSpheres™ Streptavidin-Labeled Microspheres, 0.04 µm, 488/645, 0.5% solids, ThermoFisher Scientific) and quantum dots (QDot™ 705 Streptavidin Conjugate, ThermoFisher Scientific) with dichroic mirror (AT655DC) and emission filter (LP 590, AT705/30 m). Piezostages (M-687, P-545, Physik Instrument, Germany) were used to control the defocus length. Defocused fluorescence images of polystyrene beads and quantum dots were captured with an exposure time 0.1 and 1 s, respectively, and collected in a series.

### Analytical calculation of defocused quantum dot

A defocused pattern of quantum dots on bare substrates can be obtained analytically assuming emission of quantum dots as a superposition of three linear perpendicular dipole emitters with different intensity. The orientation of dipoles can be defined by the Euler angle ($\theta ,\varphi ,\omega$). If the intensity distribution of three perpendicular dipoles with unit emission strength is assumed as *I*_1_, *I*_2_, and *I*_3_, the final intensity distribution becomes *P* = (1 − *κ*)[*I*_1_(1 + *η*)/2 + *I*_2_(1 − *η*)/2] + *κI*_3_, where the intensity ratio of the three dipoles can be defined by two parameters (*η, κ*). The far-field pattern of each dipole can be obtained by calculating the energy flux component perpendicular to the detector plane^37^.

### Pattern matching

For matching defocused quantum dot images, we adopted and modified an single-molecule image analysis algorithm based on the least-square method^39^. We selected and cropped an area in 33 × 33 pixels corresponding to 2.64 μm × 2.64 μm from an experimental defocused quantum dot image consisting of 512 × 512 pixels (40.96 μm × 40.96 μm). *R* template images (*T*^1^, *T*^2^ $\cdots$*T*^*R*^) were simulated by calculation of defocused patterns of a quantum dot while varying parameters. For each template image, two parameters ${c}_{{mn}}^{r}$ and ${d}_{{mn}}^{r}$ are calculated to minimize least-square error ${e}_{{mn}}^{r}$, where ${e}_{{mn}}^{r}=\mathop{\sum }\nolimits_{j=-16}^{16}\mathop{\sum }\nolimits_{k=-L}^{L}{s}_{{jk}}{({x}_{m+j,n+k}-{c}_{{mn}}^{r}{p}_{{jk}}^{r}-{d}_{{mn}}^{r}{b}_{{jk}})}^{2}$. Note that ${x}_{{jk}}$ denotes the pixel value with coordinate (*j,k*) in the experimental image and ${p}_{{jk}}^{r}$ is the pixel value in template images with $1\le r\le R$. Also, ${s}_{{jk}}$ and ${b}_{{jk}}$ are assumed as a disk of 13-pixel radius for supporting matrix to restrict the subarea for analysis and uniform background pattern, respectively. ${e}_{{mn}}^{r}$ can be calculated with two optimized parameters of ${c}_{{mn}}^{r}$ and ${d}_{{mn}}^{r}$. ${e}^{r}$ and ${c}^{r}$ can be obtained for each template image, where ($\widetilde{m},\widetilde{n}$) = $\mathop{{\rm{argmin}}}\limits_{(m,n)}{e}_{{mn}}^{r}$, ${e}^{r}$ = ${e}_{{\tilde{m}}{\tilde{n}}}^{r}$ and ${c}^{r}={c}_{{\tilde{m}}{\tilde{n}}}^{r}$. Then a template image (${T}^{\widetilde{r}})$ is selected as a pattern matched to an experimental image, where $\widetilde{r}=\mathop{{\rm{argmin}}}\limits_{(r)}{e}^{r}$ and ${m}_{s}=\,{c}^{\widetilde{r}}/\sqrt{{e}^{\widetilde{r}}}$. Higher *m*_s_ represents more similar images. *m*_s_ here was used as similarity index to measure pattern matching performance.

### Supplementary information

Supporting Information
