# Supplementary material for: Defocused imaging-based quantification of plasmon-induced distortion of single emitter emission
Source: Light Sci Appl. 2023 Sep 18;12:221. doi: 10.1038/s41377-023-01237-9 (PMC10505609; doi:10.1038/s41377-023-01237-9)
Supplement: Supplementary file 1 — Supporting Information [file 41377_2023_1237_MOESM1_ESM.docx]

**Supplementary Information for**

**Defocused imaging-based quantification of plasmon-induced distortion of single emitter emission**

**Gwiyeong Moon^[[1]](#footnote-2)^, Taehwang Son^[[2]](#footnote-3)^, Hajun Yoo, Changhun Lee^[[3]](#footnote-4)^, Hyunwoong Lee, Seongmin Im and Donghyun Kim***

School of Electrical and Electronic Engineering

Yonsei University, Seoul, Korea, 03722

**Figure S1. Brightfield image of nanodisk and fluorescence image of beads**

**Figure S2. AFM images of nanodisk (D_1_) and fluorescent bead (B_1_)**

**Figure S3. Defocused images of fluorescent bead on the bare substrate**

**Figure S4. AFM images of nanodisk (D_2_) and fluorescent bead (B_2_)**

**Figure S5. Defocused images of dipole (*λ* = 645 nm) on bare substrate**

**Figure S6. Comparison of intensity according to the orientation of dipole**

**Figure S7. Brightfield image of nanodisk and fluorescence image of quantum dot**

**Figure S8. AFM image of nanodisk (D_3_)**

**Figure S9. Defocused images of a quantum dot on the bare substrate (*f*** $\boldsymbol{\approx}$ **-0.9 μm)**

**Figure S10. Defocused images of a fluorescence bead on the bare substrate (*f*** $\boldsymbol{\approx}$ **-1.1 μm)**

**Figure S11. AFM images of a nanodisk (D_4_) and its profile**

**Figure S12. Subset of simulated template images for matching a defocused quantum dot image**

**Figure S13. Subset of the experimental defocused image of QD_ref68-203_ with *f*** $\boldsymbol{\approx}$ **-0.6 μm**

**Figure S14. Simulate defocused images of dipole (y and z axis) near nanodisk and on the bare substrate**

**Figure S15. Power flow in the emitter-antenna system**

**Figure S16. Defocused images of a dipole in the three orthogonal orientations for *λ =* 421 - 900 nm with *d* = 10 nm**

**Figure S17. Process and parameters for estimation of distance using defocused images of a dipole**

**Figure S18. Structural similarity index measure (SSIM) between defocused images of *d* < 150 nm and reference (*d* = 150 nm) for each of the three orthogonal dipoles**

**Figure S19. Estimation of dipole orientation with pattern matching method**

**Figure S20. Mislocalization for localization with focused images, pattern matching of defocused images, and localization and pattern matching (LPM)**

**Text S1. Coupled dipole theory for an emitter-nanosphere system**

**Table S1. Summarized parameter values for the simulation of defocused quantum dot images**

**Figure S1**

**Bright field image of nanodisks and fluorescence image of beads**


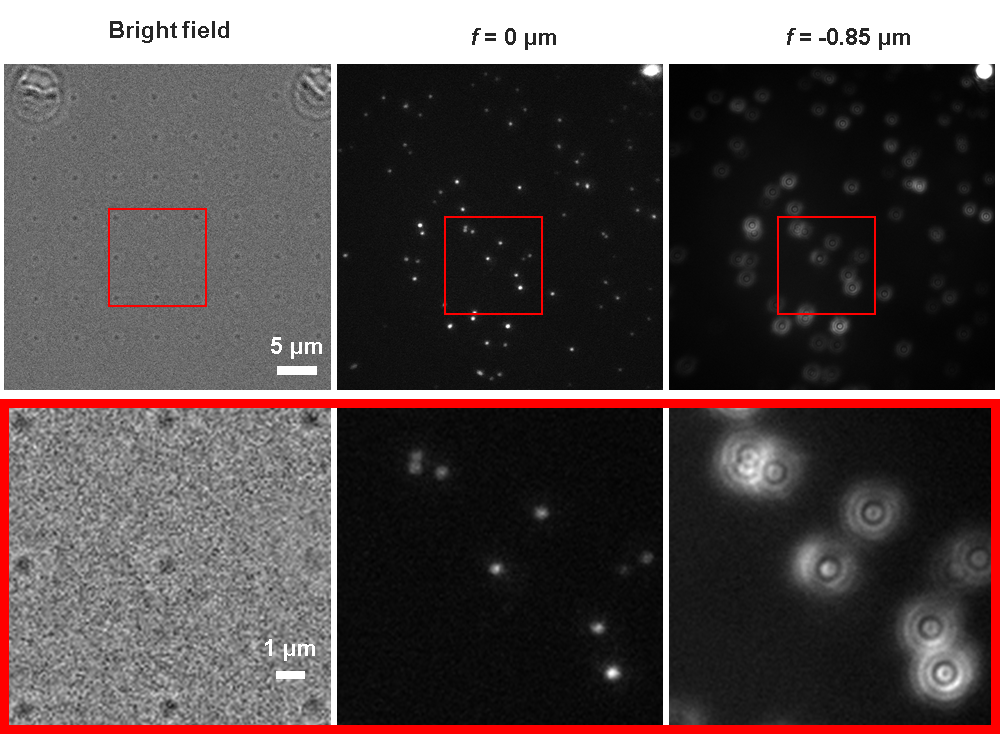


**Figure S1** The whole field-of-view of the bright field and fluorescence images is presented. Only a specific area was cropped to analyze the defocused patterns of a fluorescence bead in the proximity to a nanodisk that are described in the main text.

**Figure S2**

**AFM images of nanodisk (D_1_) and fluorescent bead (B_1_)**

**
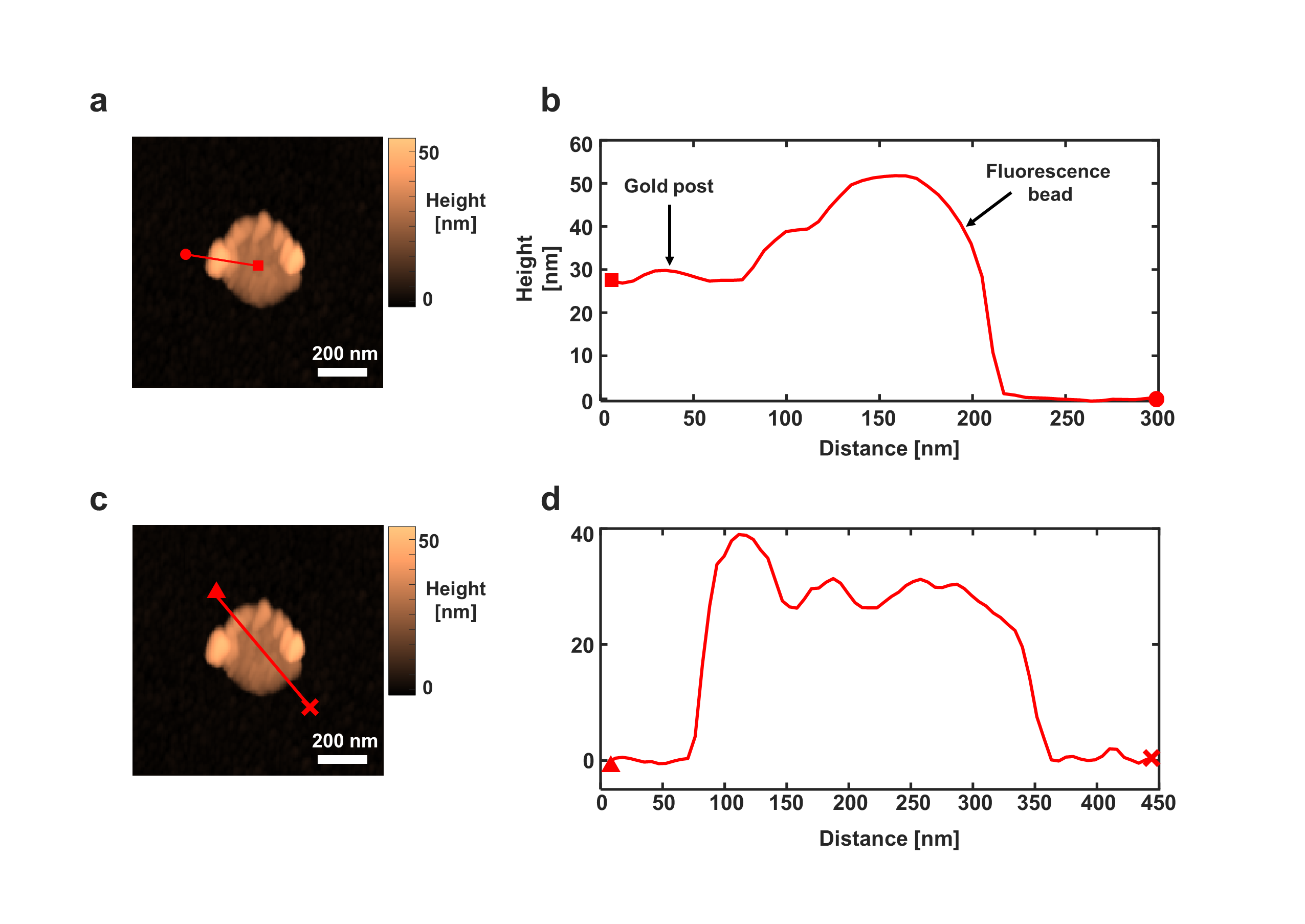
**

**Figure S2** AFM image of nanodisk and fluorescence bead and the height profile. (a,b) The height profile on the left-hand side of the nanodisk (D_1_) between square and circle show a bump that is correlated with the fluorescent bead (B_1_). The data confirm existence of two beads on the right and the left side of D_1_. Comparison with the bright-field and the fluorescence image provided in Figure 2(a) shows that fluorescence of B_1_ is correlated with the bead on the left. The bump on the right may be associated with a fluorescently quenched bead. (c,d) The height profile across D_1_ showing the diameter of D_1_ as 295 nm.

**Figure S3**

**Defocused images of fluorescent bead on the bare substrate**

**
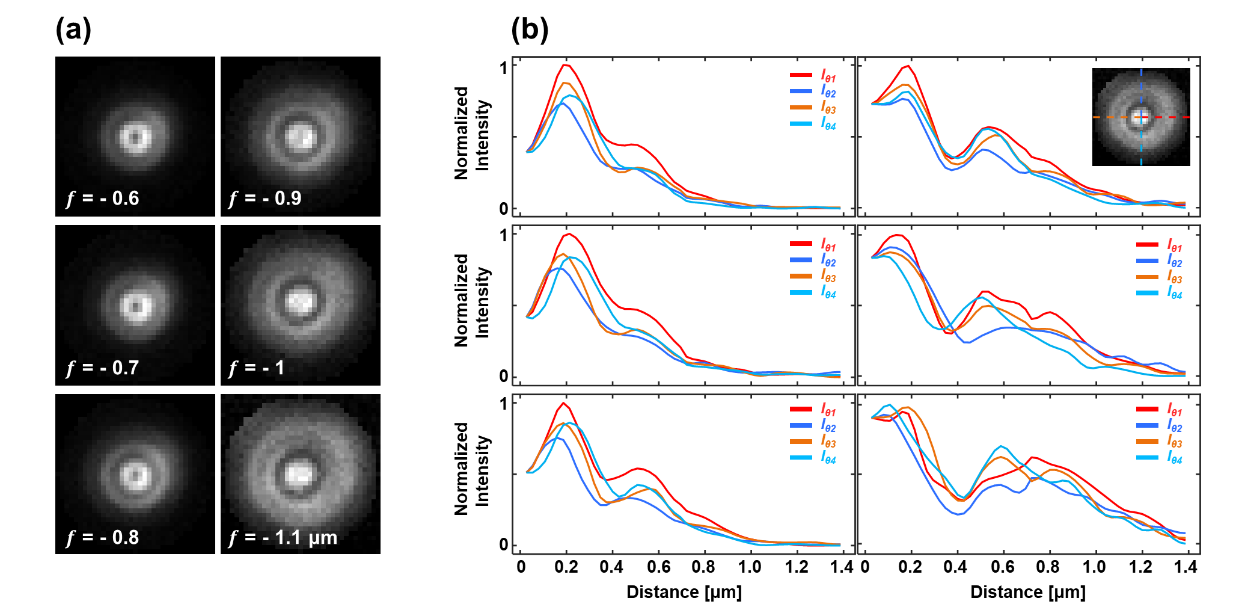
**

**Figure S3** (a) Defocused images of a fluorescent bead in the absence of a nanodisk. Defocusing was conducted by moving the objective lens by 0.6 ~ 1.1 μm toward the fluorescent bead. (b) The intensity profile in the four directions (*θ* = 0º, 90º, 180º, and 270º) from the image center.

The defocused fluorescence intensity of a fluorescent bead in the absence of a nanodisk was measured as a reference for comparison with a fluorescent bead in the vicinity of a nanodisk. Figure S3(a) shows the reference fluorescence images measured with a defocus value of *f* = -0.6, -0.7, -0.8, -0.9, 1, and 1.1 μm. *f* represents how much an image is defocused, measured by the displacement of an objective lens to make a defocused fluorescence image. *f* = 0 corresponds to the focal plane located at the axial position of an emitter. A defocused image of a fluorescent bead shows circular symmetry consisting of concentric gaussian rings with an identical center [1]. Defocused fluorescent intensity profiles along the four orthogonal directions (*θ* = 0º, 90º, 180º, and 270º) are plotted in Figure S3(b) and showed an almost identical trend in each case of *f*, suggesting circular symmetry.

(1) Speidel, M.; Jonáš, A.; Florin, E.-L. *Opt. Lett.* **2003,** 28, (2), 69-71.

**Figure S4**

**AFM images of nanodisk (D_2_) and fluorescent bead (B_2_)**

**
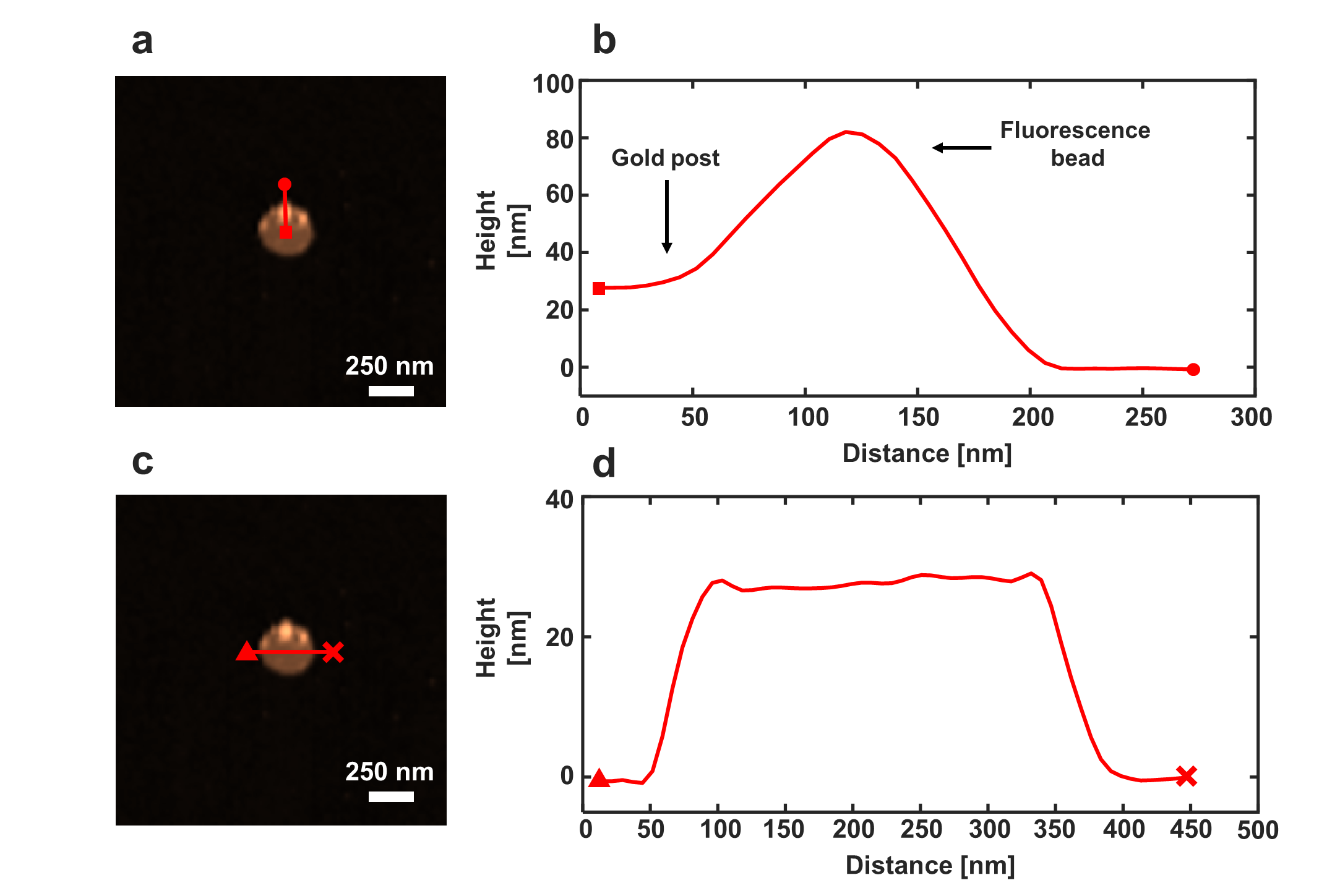
**

(a), (c) The AFM images of nanodisk (D_2_) and fluorescence bead (B_2_) (b) The height profile from point of square to circle is plotted showing that fluorescence bead (B_2_) was located on the nanodisk (D_2_). (d) The height profile showing the diameter of nanodisk.

**Figure S5**

**Defocused images of dipole (λ = 645 nm) on bare substrate**

**
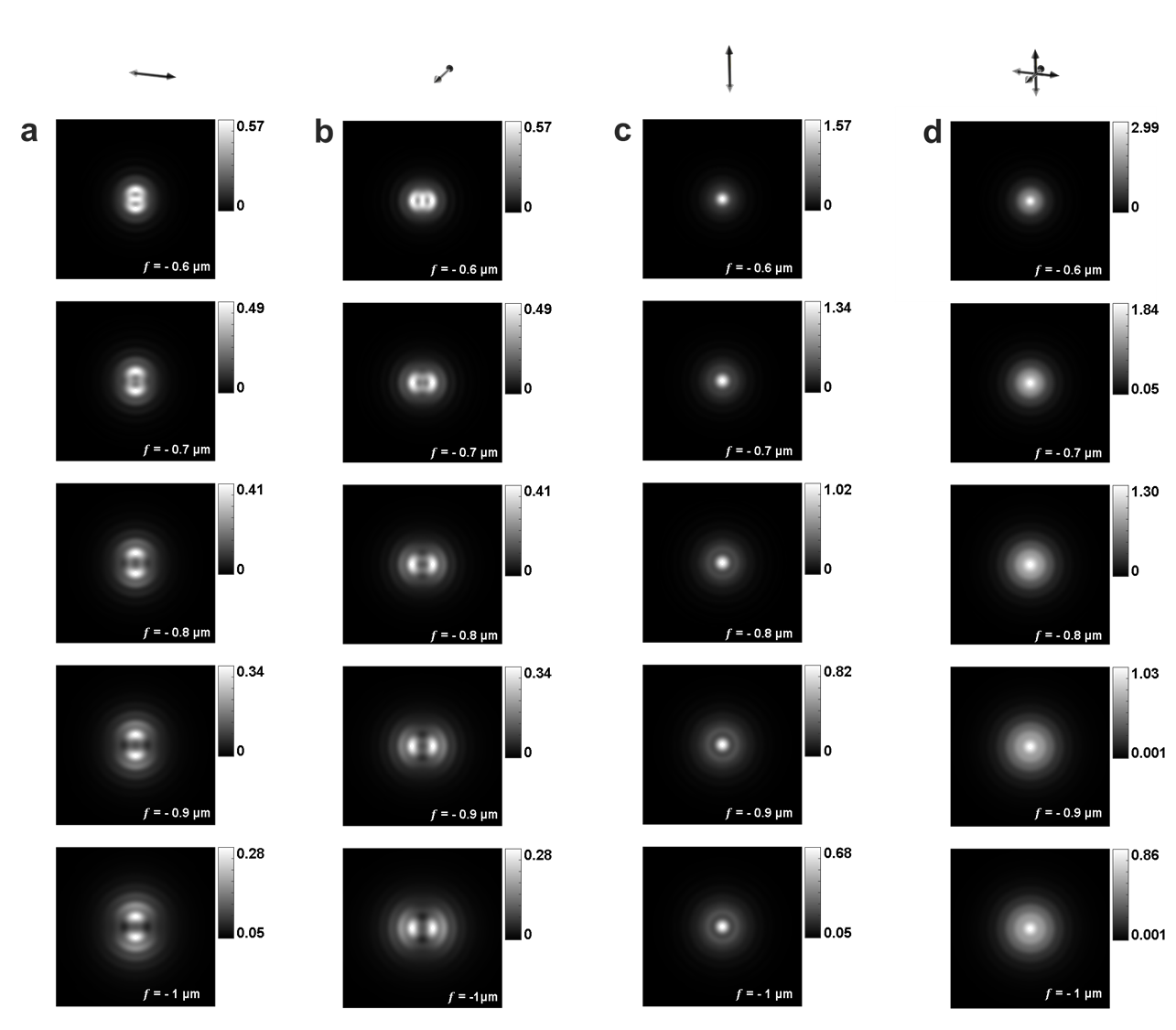
**

The defocused pattern (f = -0.6, -0.7, -0.8, -0.9, 1 μm) of dipole (*λ* = 645 nm) which is oriented (a) x-axis, (b) y-axis, and (c) z-axis. (d) The incoherent sum of defocused pattern of three dipoles.

**Figure S6**

**Comparison of intensity according to the orientation of dipole**


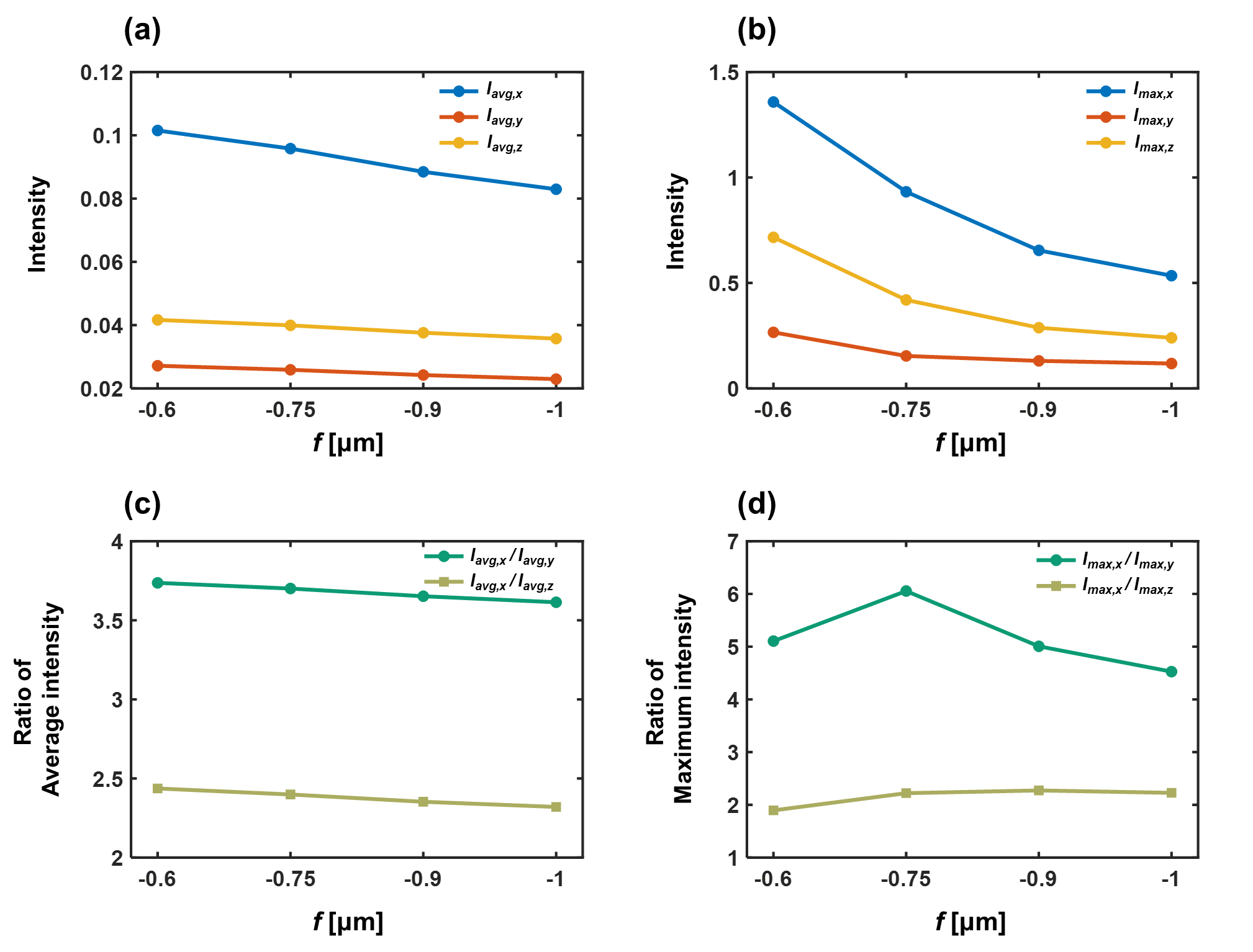


The maximum and average intensity of defocused images with *f* = -0.6, -0.75, -0.9, and -0.1 μm in Figure 2(g-i) are plotted in Figure S6(a) and (b). The x-axis oriented dipole makes much larger the average and maximum intensity of defocused image than other axis oriented dipoles. The average and maximum intensity of x-pol and y-pol/z-pol are presented in Figure S6(c) and (d). Note that the nanodisk and dipole are located on the x-axis.

**Figure S7**

**Brightfield image of nanodisk and fluorescence image of quantum dot**

**
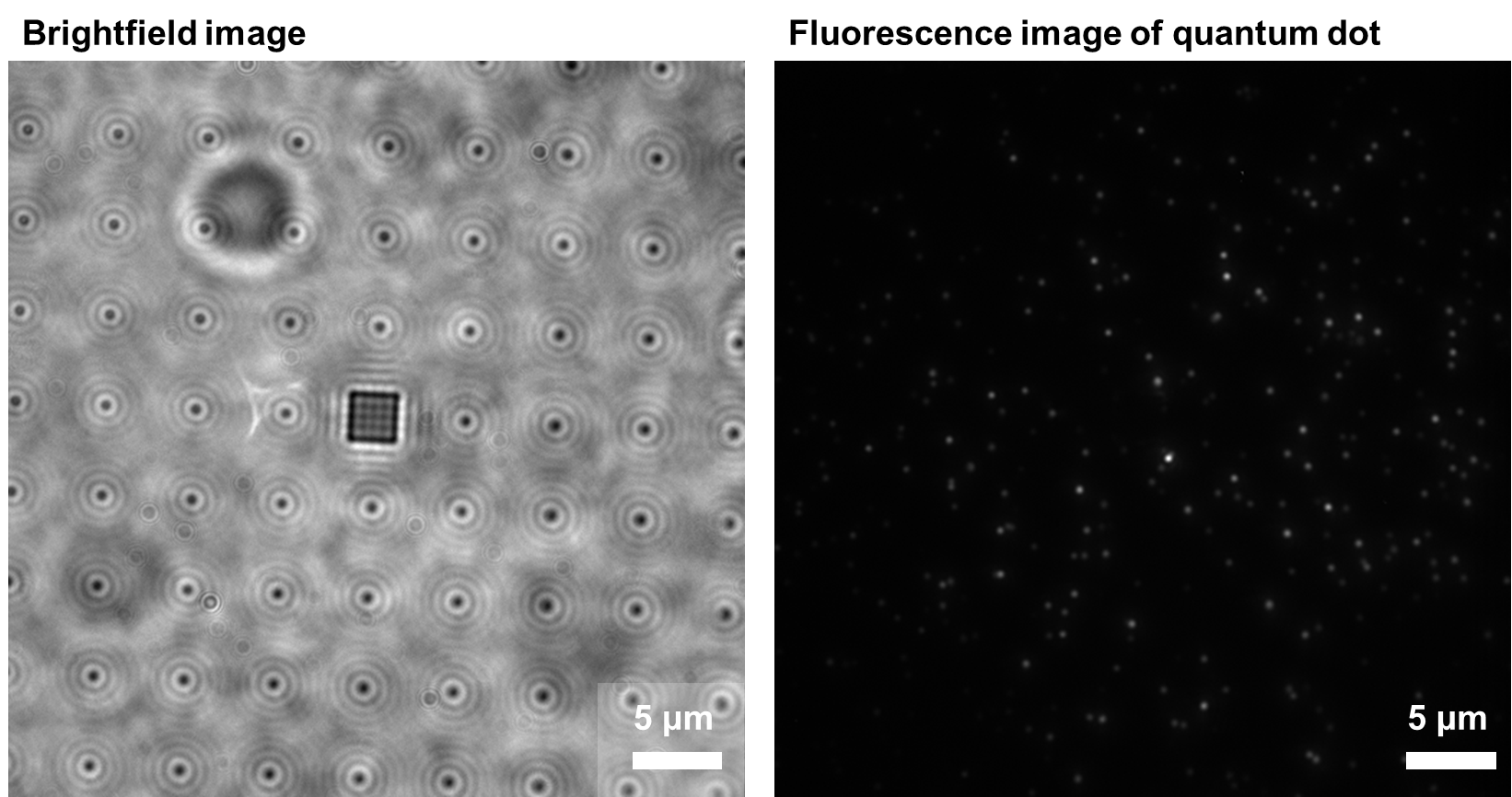
**

The whole field-of-view of the bright field and fluorescence image of quantum dots. The specific area was cropped to analyze the defocused pattern of quantum dots close to a nanodisk described in the main text.

**Figure S8**

**AFM image of nanodisk (D_3_)**

**
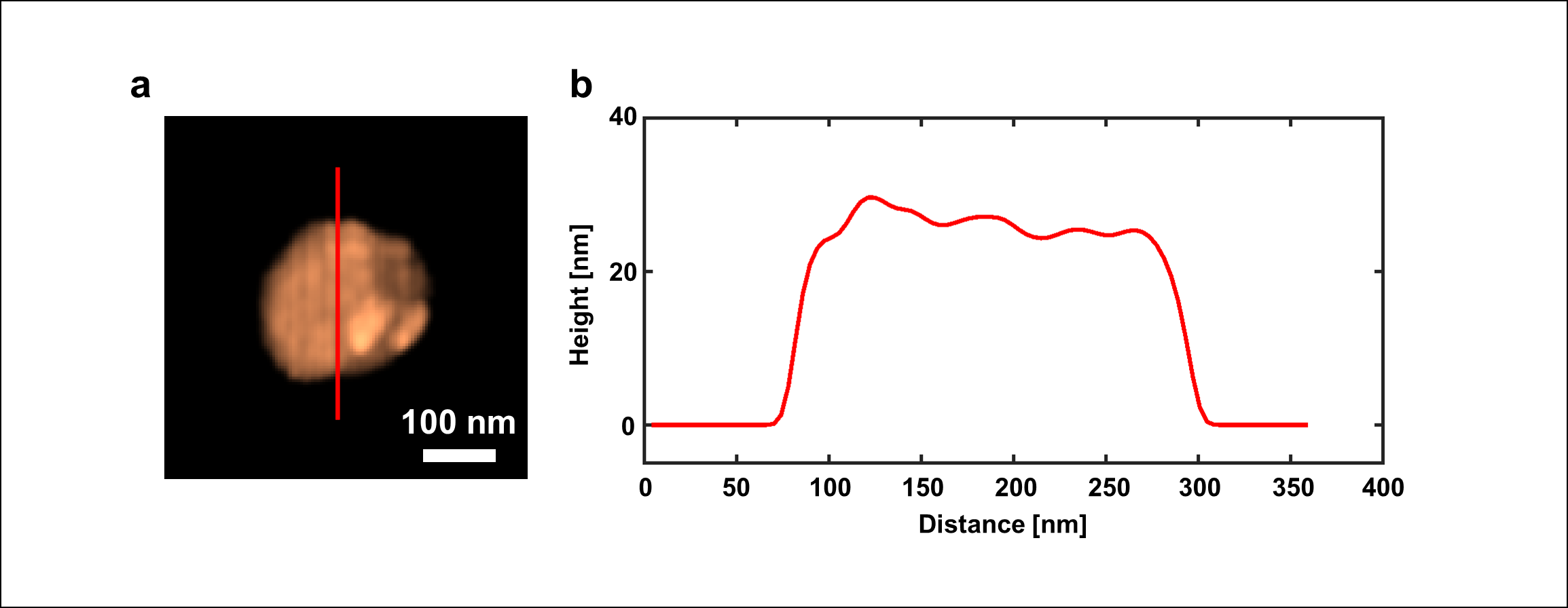
**

(a) The AFM images of nanodisk (D_3_). (b) The height profile across the red line of (a).

**Figure S9**

**Defocused images of a quantum dot on the bare substrate (*f*** $\boldsymbol{\approx}$ **-0.9 μm)**

**
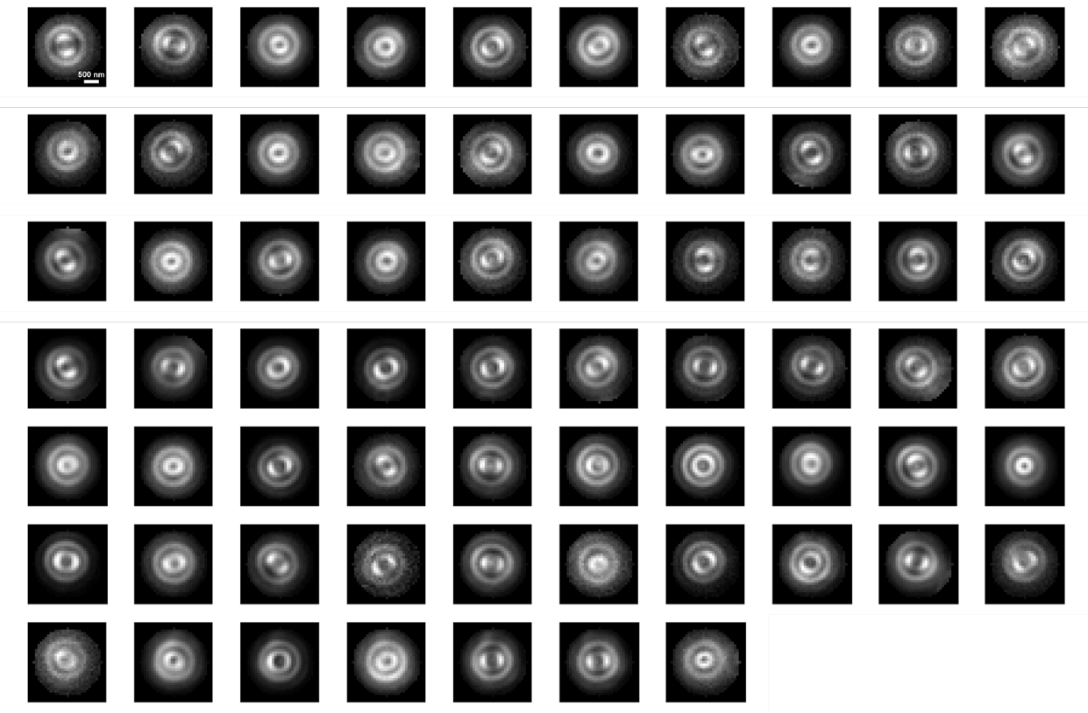
**

Experimentally obtained defocused images (*f* $\approx$ -0.9 μm) of quantum dots on the bare glass substrate. The nature of quantum dots as an anisotropic emitter generated diverse defocused patterns in contrast to fluorescence beads which is an isotropic emitter.

**Figure S10**

**Defocused images of a fluorescence bead on the bare substrate (*f*** $\boldsymbol{\approx}$ **-1.1 μm)**

**
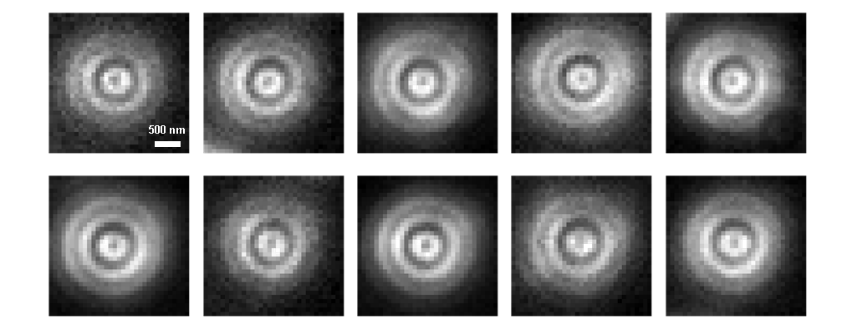
**

Defocused images at *f* = -1.1 μm of 40-nm fluorescent beads on the bare glass substrate. Almost identical defocused patterns can be observed because of isotropic emission characteristics.

**Figure S11**

**AFM images of a nanodisk (D_4_) and its profile**

**
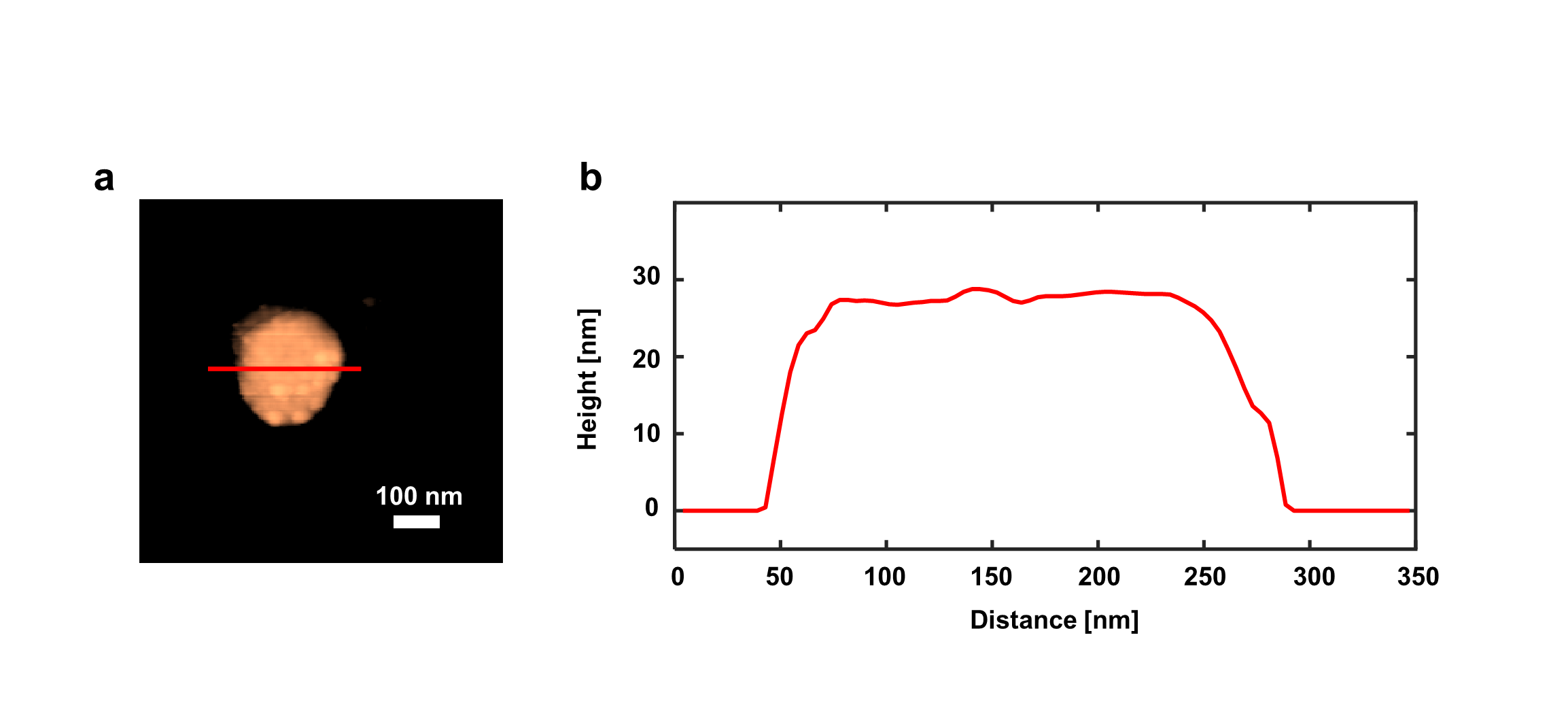
**

(a) The AFM images of nanodisk (D_4_). (b) The height profile across the red line of (a).

**Figure S12**

**Subset of simulated template images for matching a defocused quantum dot image**

**
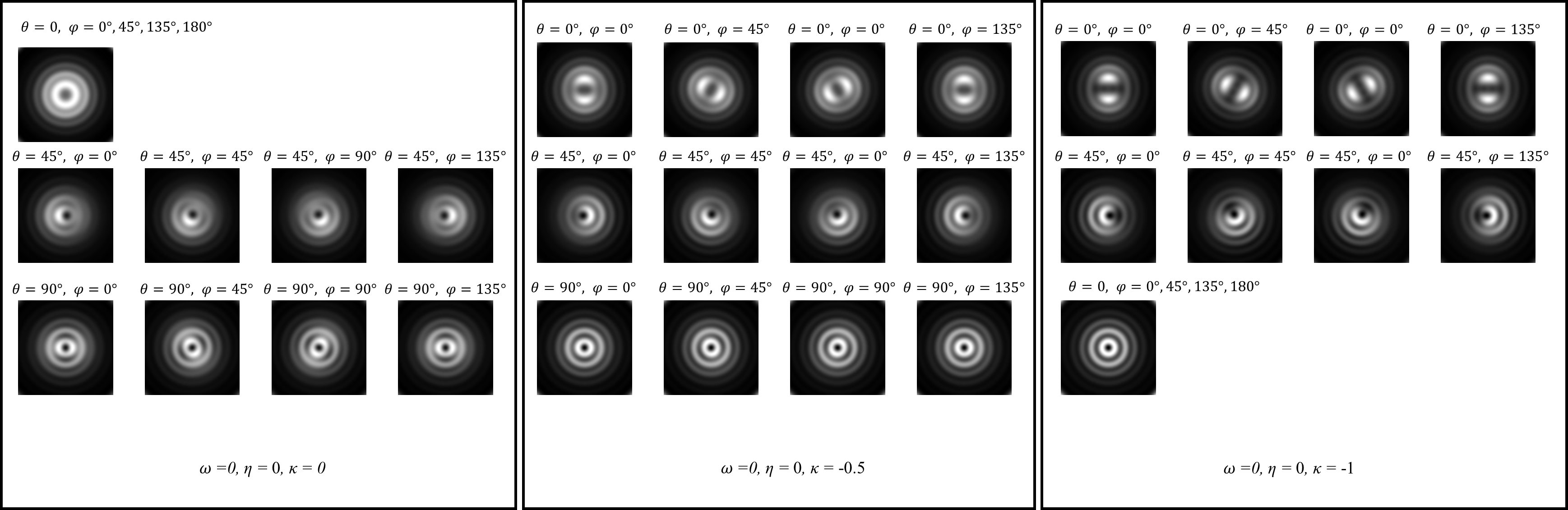
**

Orientation of emitter dipoles caused significant changes in defocused images. The orientation of a dipole can be represented by variables of Euler angle ($\theta, \varphi, \omega$) and intensity ratio (*η, κ*) to describe three perpendicular dipoles.

**Figure S13**

**Subset of the experimental defocused image of QD_ref68-203_ with *f*** $\boldsymbol{\approx}$ **-0.6 μm**


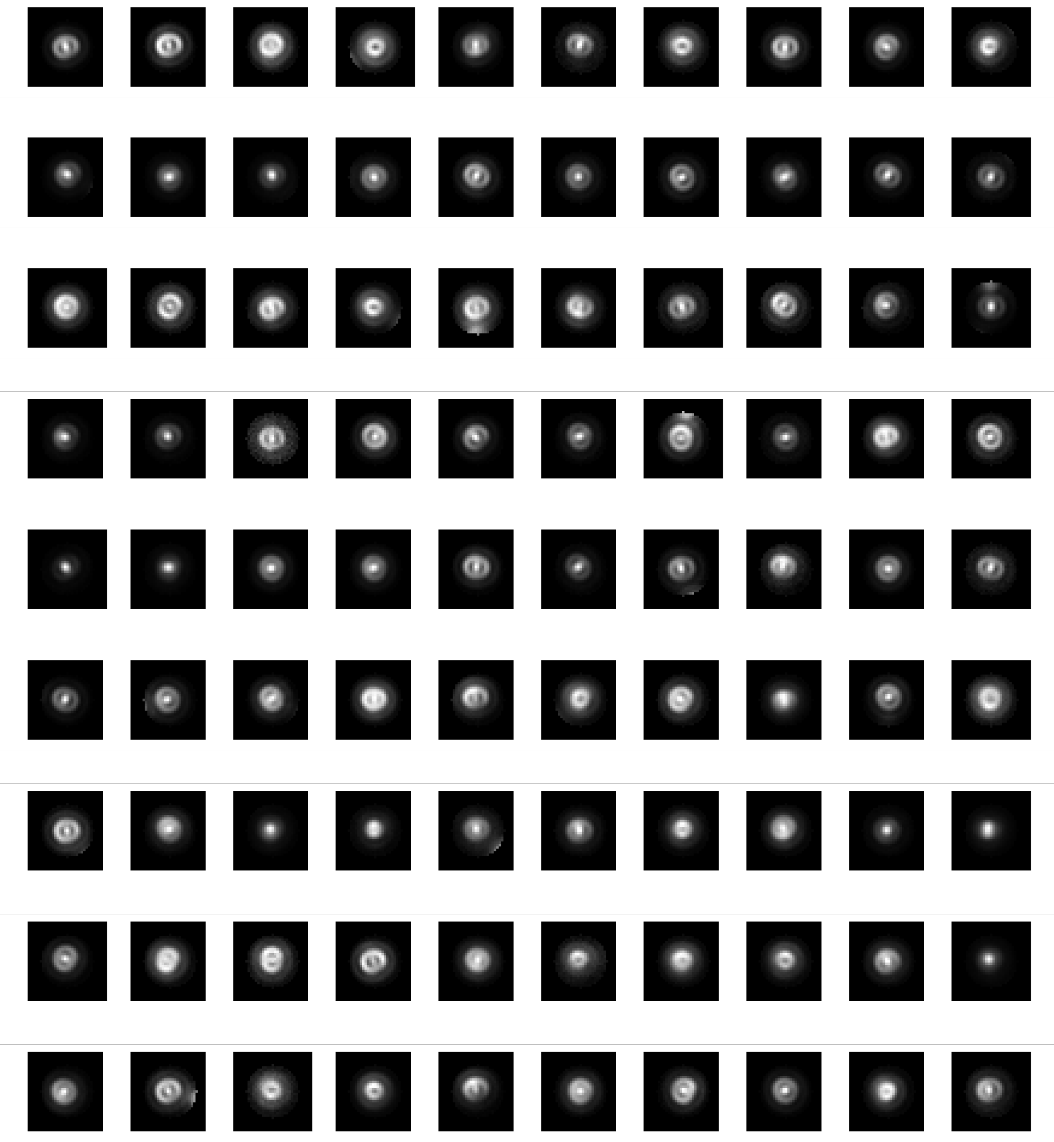


**Figure S14**

**Simulate defocused images of dipole (y- and z- axis) near nanodisk and on the bare substrate**


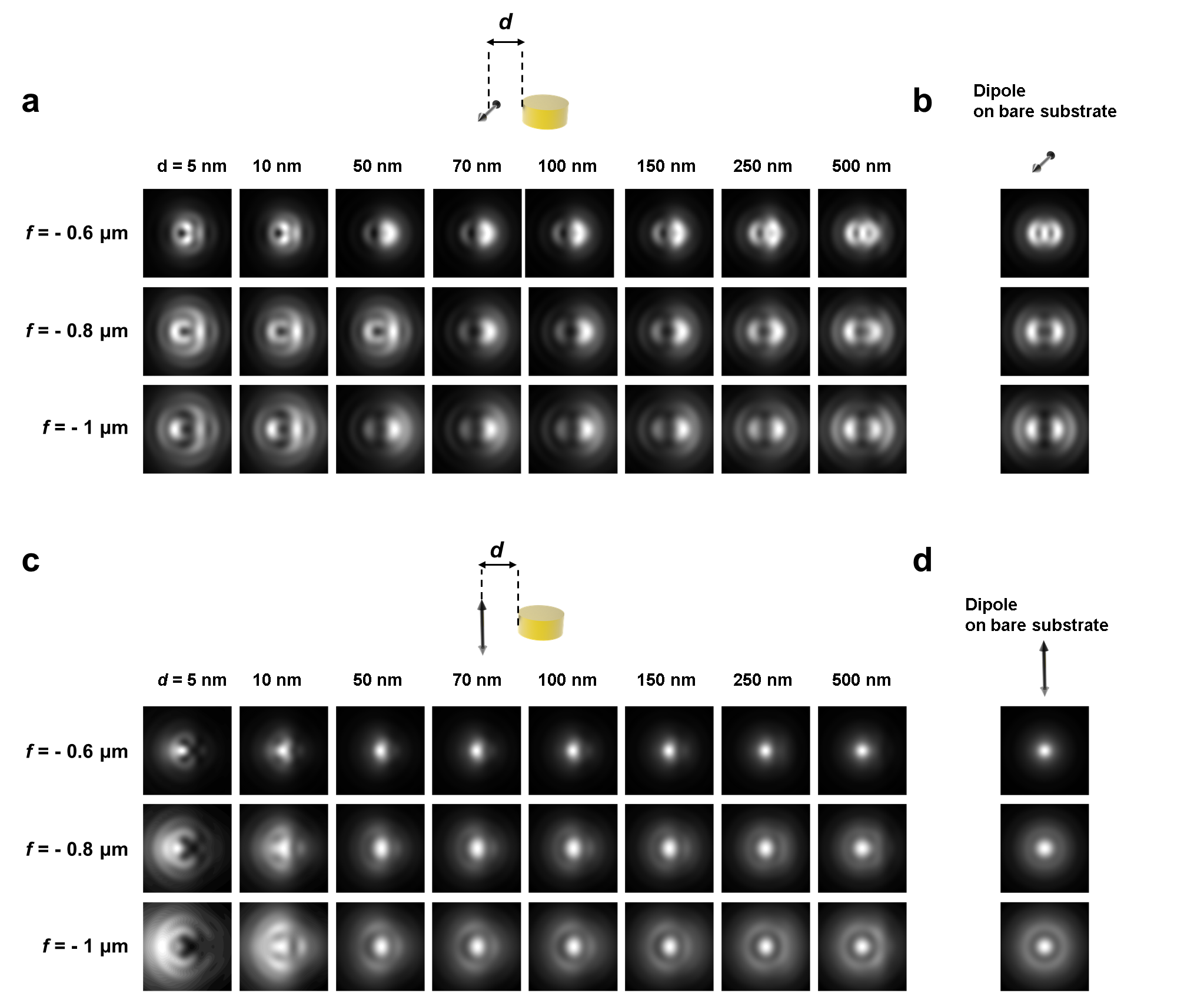


**Figure S14** Calculated defocused far field patterns: (a) a dipole is oriented along the y-axis in the vicinity of a nanodisk. (b) A dipole oriented along the y-axis on the bare substrate. (c) A dipole oriented along the z-axis in the vicinity of nanodisk. (d) A dipole oriented along the z-axis on the bare substrate.

The defocused far-field patterns of the dipoles oriented in the y and z-axis was simulated by FDTD calculation for the two cases: in the vicinity of nanodisk and on the bare substrate. The defocused patterns with *d* = 500 nm are almost identical to those of a dipole on the bare substrate.

**Figure S15**

**Power flow in the emitter-antenna system**

**
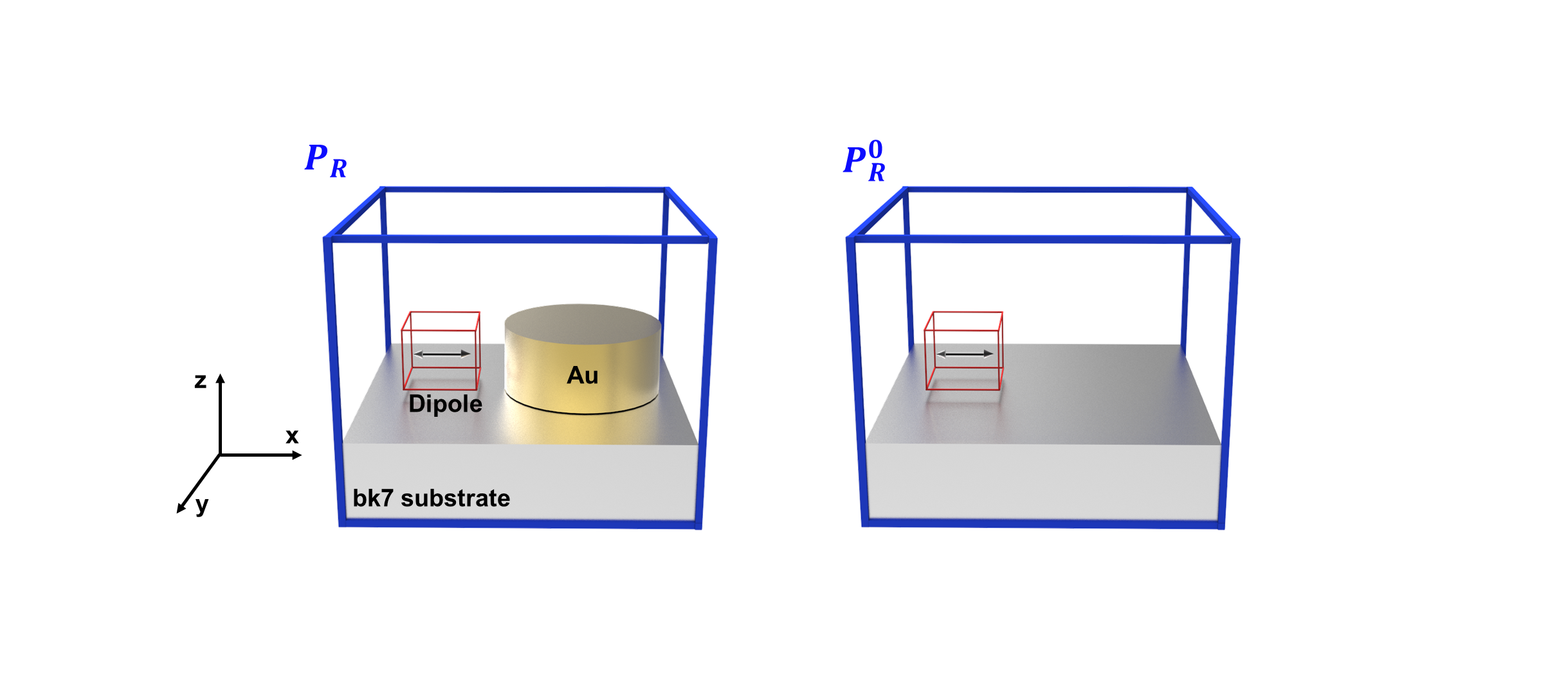
**

The power flow in an emitter-antenna system was calculated using FDTD simulation. A 270-nm diameter gold nanodisk was placed on the BK7 substrate. The distance between gold nanodisk and the dipole center (wavelength = 705 nm) was varied from 5 nm to 100 nm. The orientation of a dipole was assumed to be in the three Cartesian coordinate directions. Power radiated into the far-field in the presence of the nanodisk (*P_R_*) and in its absence ($P_{R}^{0}$) is measured by calculating the net power through each blue box. Radiated power enhancement was calculated by *R* = *P_R_* /$P_{R}^{0}$.

**Figure S16**

**Defocused images of a dipole in the three orthogonal orientations for λ = 421 - 900 nm with *d* = 10 nm**

**
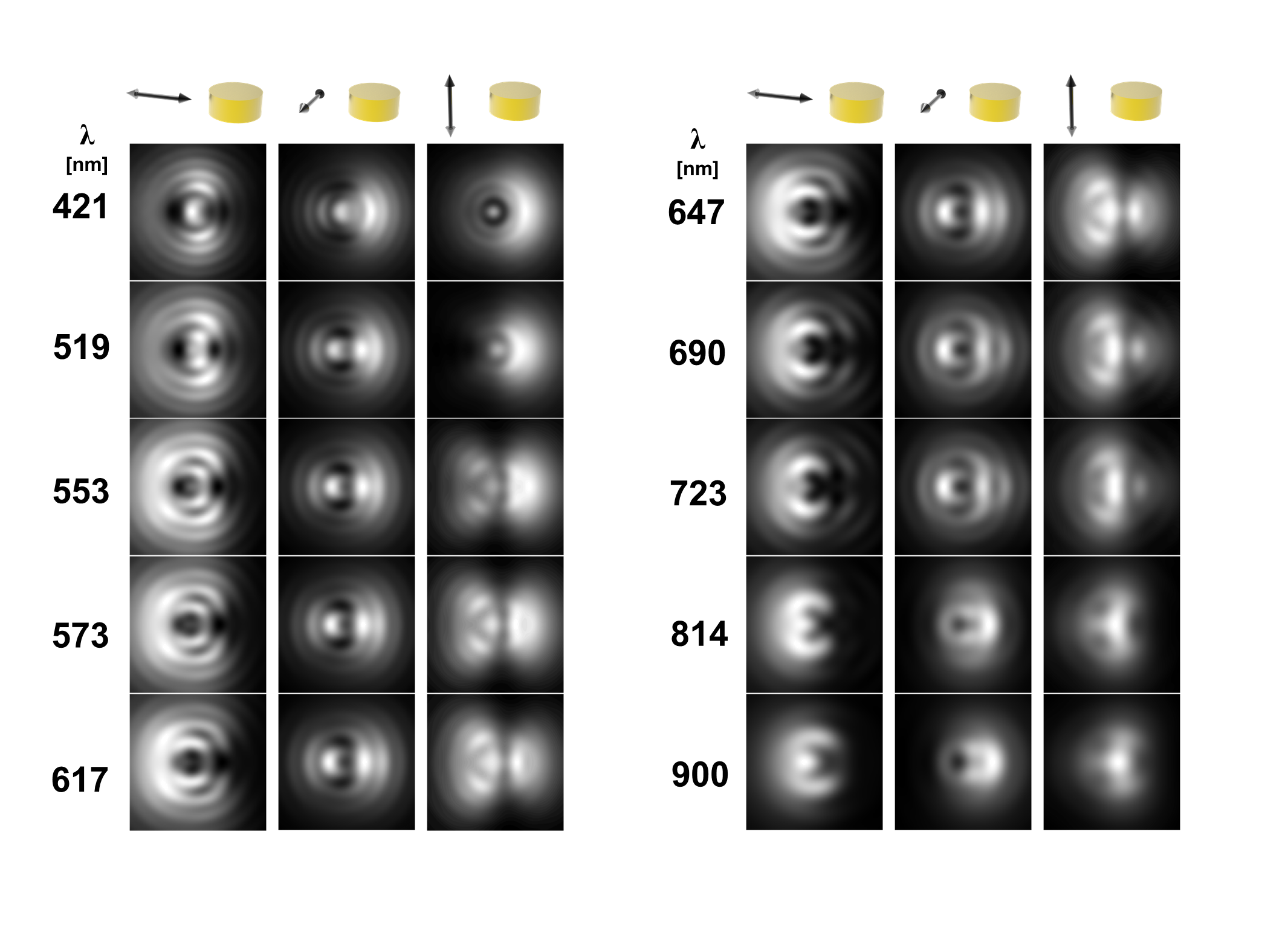
**

**Figure S17**

**Process and parameters for estimation of distance using defocused images of a dipole**

**
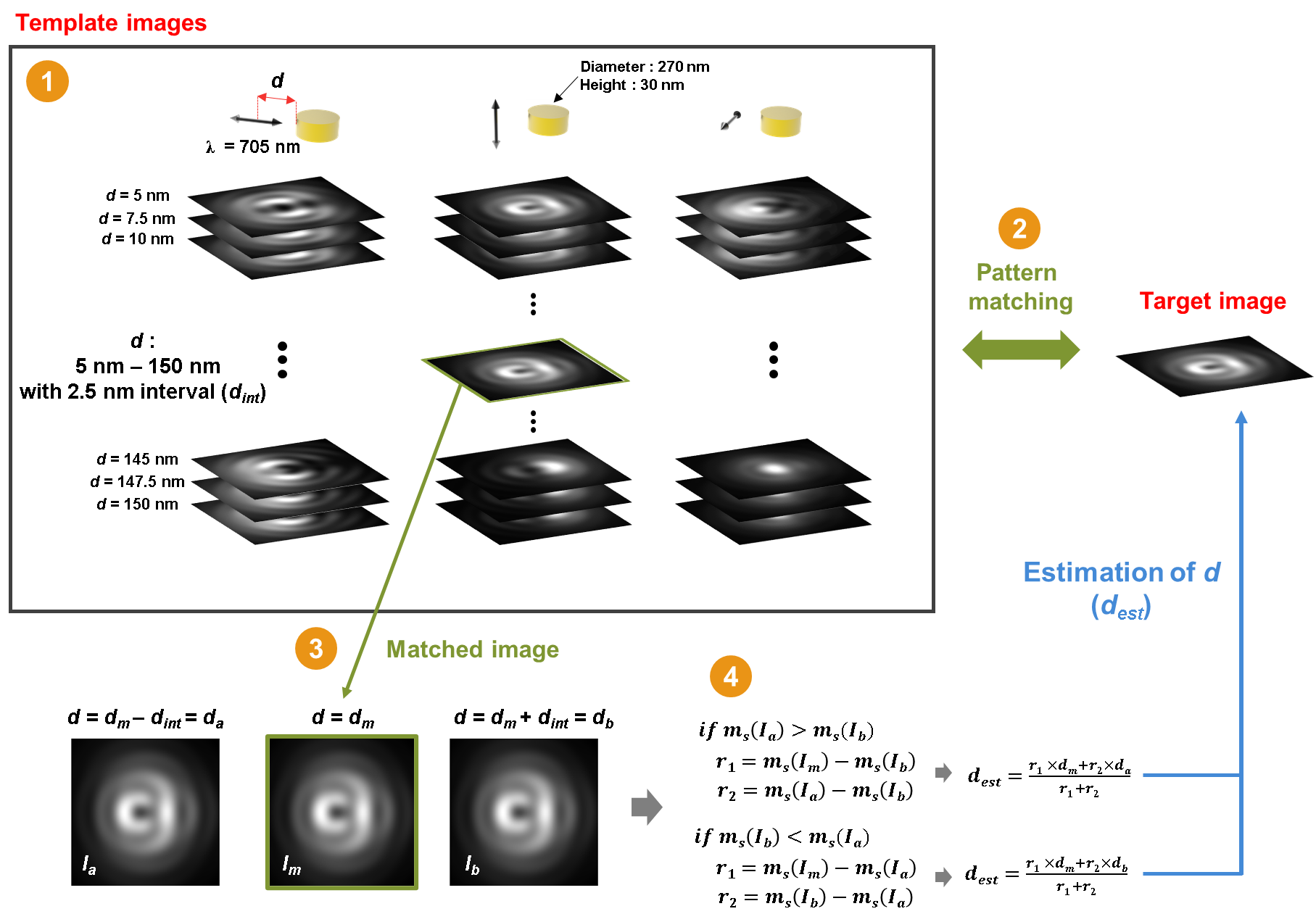
**

For estimation of the distance between a dipole and the nanodisk using defocused images, the template was formed using FDTD simulation. The diameter and height of a nanodisk was assumed as 270 nm and 30 nm, respectively. Three orthogonal orientations of a dipole were assumed with emission wavelength of 705 nm. The distance between nanodisk and dipole was varied from 5 nm to 150 nm. A target image was obtained with distance which was randomly chosen in the range of the distance and pattern matching was conducted with the template images. As a result of pattern matching, when the distance of a dipole in the matched image (*I_m_*) is *d_m_*, we estimate the distance using *m_s_* of images which correspond to one interval from *d_m_*, i.e., *d_m_* – *d_int_* and *d_m_* + *d_int_*. *d_est_* was determined by calculation of internal division of *d_m_* and distance corresponding to a higher value of *m_s_* between *d_m_* – *d_int_*, *d_m_* + *d_int_*. The ratio (*r_1_* and *r_2_*) of the internal division is presented in Figure S17.

**Figure S18**

**Structural similarity index measure (SSIM) between defocused images of *d* < 150 nm and reference (*d* = 150 nm) for each of the three orthogonal dipoles**

**
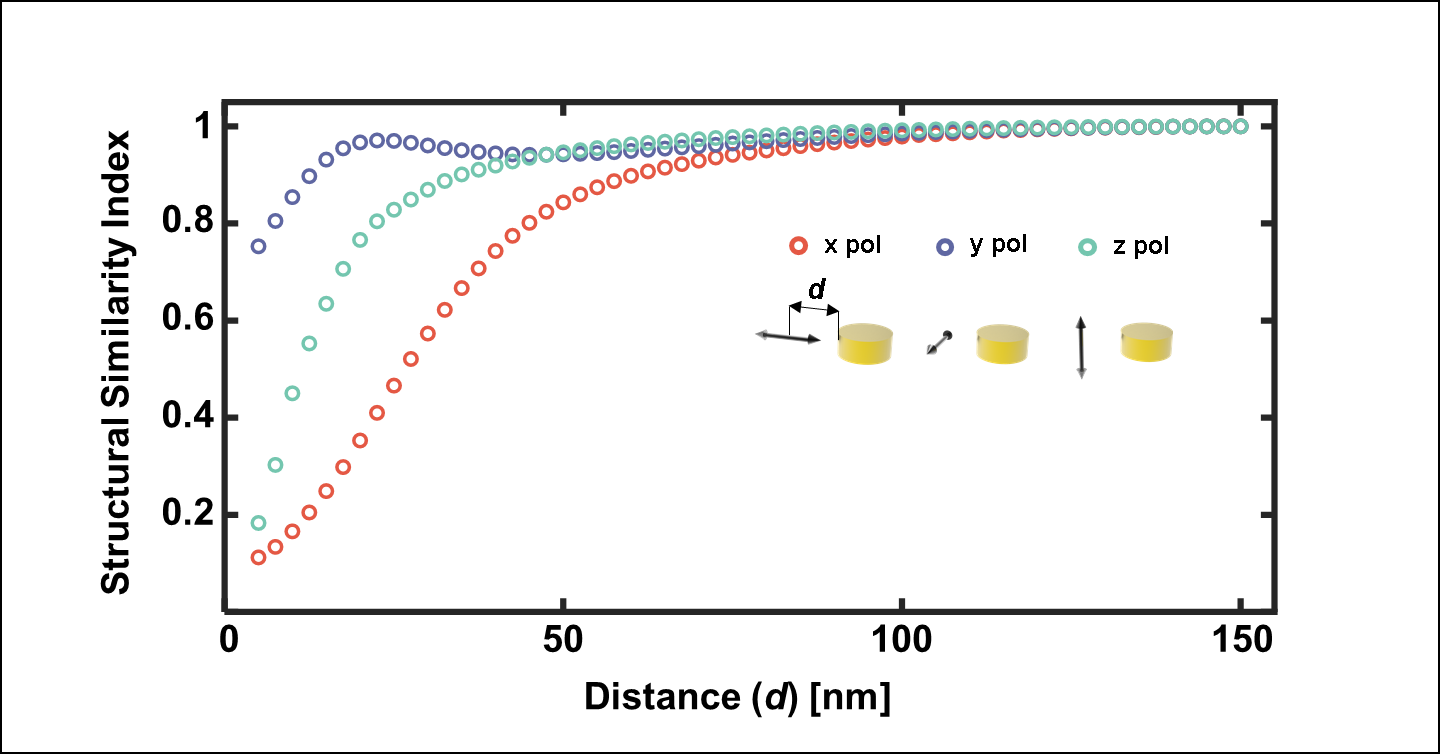
**

SSIM for defocused images of dipoles oriented in the *x*-axis tends to be lower than that of dipoles aligned in the y and z axis. It is suggested that the defocused images of dipoles in the *x*-axis vary more significantly with the distance between dipole and nanodisk. The result may be associated with the results of pattern matching of defocused images.

**Figure S19**

**Estimation of dipole orientation with pattern matching method**


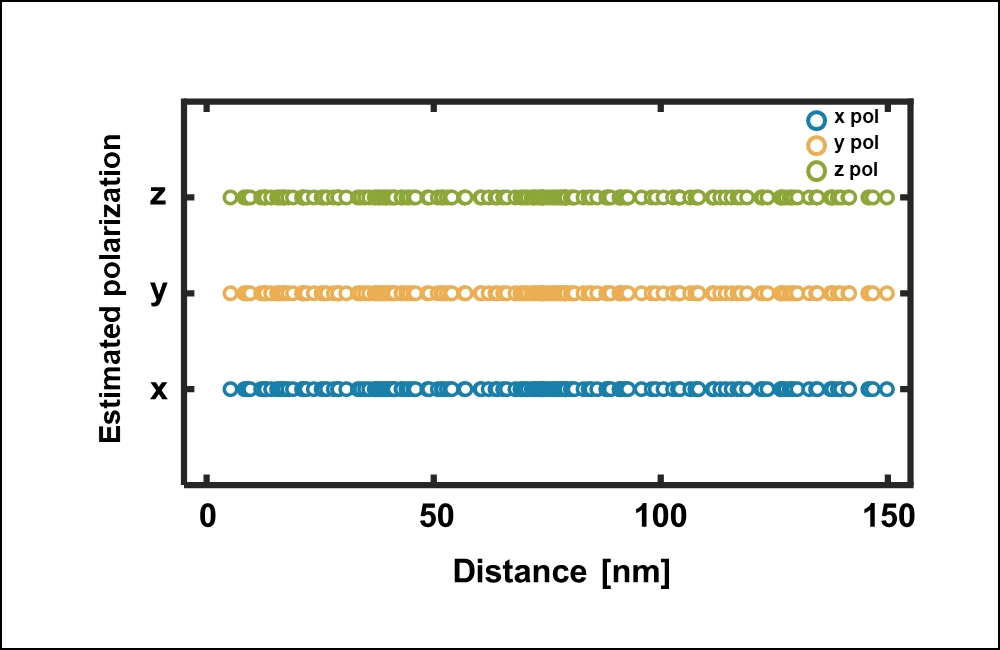


The orientation of a dipole can be perfectly estimated by pattern matching of defocused images for *d* < 150 nm.

**Figure S20**

**Mislocalization for localization with focused images, pattern matching of defocused images, and localization and pattern matching (LPM)**


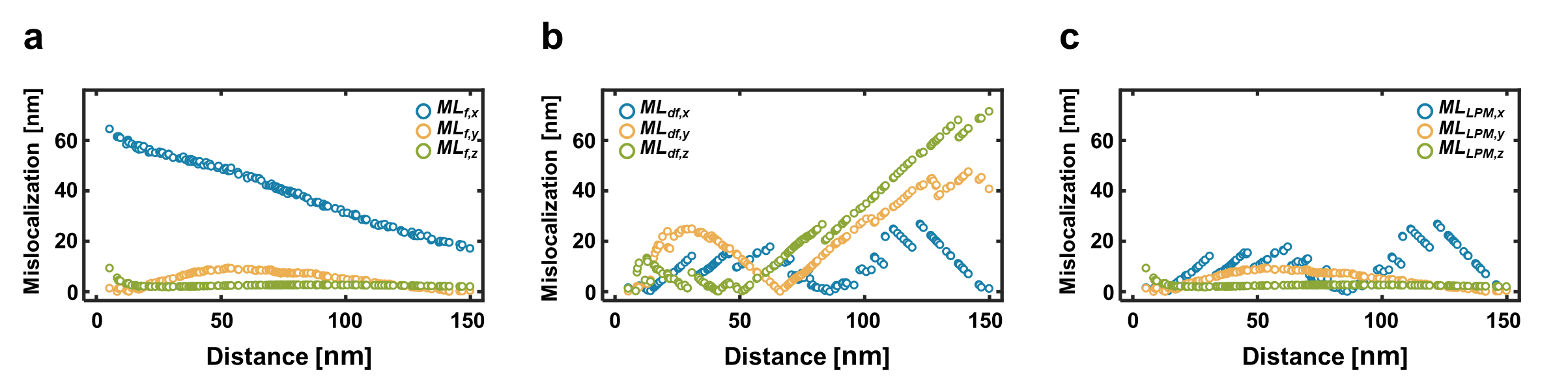


**Figure S20** Mislocalization of dipoles if localized by three methods: (a) using focused images, (b) pattern matching of defocused images, and (c) localization and pattern matching (LPM).

**Text S1**

**Coupled dipole theory for an emitter-nanosphere system**

An emitter-nanosphere system can be modeled as a pair of coupled dipole system. The dipole moment of induced polarization (**p**_1_) of the nanosphere was obtained by the following equation:

$$p_{1}= a^{3}\frac{\varepsilon\left( \omega_{0} \right)-1}{\varepsilon\left( \omega_{0} \right)+2}\frac{g(\boldsymbol{d})}{e^{2}}$$

Here, $a$ and $\varepsilon\left( \omega\right)$ denote the radius of nanosphere and bulk Drude dielectric function. The amplitude and phase of the transition dipole moment (**p**_0_) of an emitter was assumed as 1 and 0, respectively. Also, $g(\boldsymbol{d})$ was defined as the coupling strength between **p**_0_ and induced polarization of the nanosphere **p**_1_ (see details are in [1]). We assumed the resonance energy of **p**_0_ and **p**_1_ to be 2.60eV and 2.55 eV. The amplitude and phase of **p**_1_ was calculated with respect to the distance between **p**_0_ and the center of nanosphere. When *d* = 50 nm, the amplitude and phase of **p**_1_ was about 4.46 and 0.81$\pi$.

[1] Goldwyn, H. J.; Smith, K. C.; Busche, J. A.; Masiello, D. J. *ACS Photonics*, **2018**, *5*, 3141-3151.

**Table S1**

**Summarized parameter values for the simulation of defocused quantum dot images**

| Parameters | $\theta$ | $\varphi$ | $\omega$ | *η* | *κ* | *M* | *F* |
| --- | --- | --- | --- | --- | --- | --- | --- |
| Start value | 0º | 0º | 0º | -1 | 0 | 190 | -1.1 |
| End value | 90º | 180 º | 90º | 0 | 1 | 220 | -0.8 |
| Interval | 30º | 10º | 45 º | 0.25 | 0.3 | 10 | 0.1 |

The defocused patterns of a quantum dot was simulated by scanning values of the orientation ($\theta, \varphi,\omega$) of the three perpendicular dipoles and the intensity ratio (*η, κ*). Magnification (*M*) and *f* were also varied considering error that may occur in experiments. Numerical aperture of an objective lens was fixed at 1.49.

1. Current affiliation: LG Innotek, Seoul, South Korea, 07796 [↑](#footnote-ref-2)
2. Current affiliation: Center for Systems Biology, Massachusetts General Hospital, Boston, Massachusetts, USA [↑](#footnote-ref-3)
3. Current affiliation: LG Display, Paju, Gyeonggi-do, South Korea, 10845

   * Corresponding author: kimd@yonsei.ac.kr [↑](#footnote-ref-4)
